# Sequencing and *de novo *analysis of a coral larval transcriptome using 454 GSFlx

**DOI:** 10.1186/1471-2164-10-219

**Published:** 2009-05-12

**Authors:** Eli Meyer, Galina V Aglyamova, Shi Wang, Jade Buchanan-Carter, David Abrego, John K Colbourne, Bette L Willis, Mikhail V Matz

**Affiliations:** 1 University of Texas at Austin, 1 University Station C0930, Austin, TX, 78712, USA; 2 The Center for Genomics and Bioinformatics, Indiana University, 915 East Third Street, Bloomington, IN, 47405, USA; 3 ARC Centre of Excellence for Coral Reef Studies, and School of Marine and Tropical Biology, James Cook University, Townsville, QLD, 4811, Australia

## Abstract

**Background:**

New methods are needed for genomic-scale analysis of emerging model organisms that exemplify important biological questions but lack fully sequenced genomes. For example, there is an urgent need to understand the potential for corals to adapt to climate change, but few molecular resources are available for studying these processes in reef-building corals. To facilitate genomics studies in corals and other non-model systems, we describe methods for transcriptome sequencing using 454, as well as strategies for assembling a useful catalog of genes from the output. We have applied these methods to sequence the transcriptome of planulae larvae from the coral *Acropora millepora*.

**Results:**

More than 600,000 reads produced in a single 454 sequencing run were assembled into ~40,000 contigs with five-fold average sequencing coverage. Based on sequence similarity with known proteins, these analyses identified ~11,000 different genes expressed in a range of conditions including thermal stress and settlement induction. Assembled sequences were annotated with gene names, conserved domains, and Gene Ontology terms. Targeted searches using these annotations identified the majority of genes associated with essential metabolic pathways and conserved signaling pathways, as well as novel candidate genes for stress-related processes. Comparisons with the genome of the anemone *Nematostella vectensis *revealed ~8,500 pairs of orthologs and ~100 candidate coral-specific genes. More than 30,000 SNPs were detected in the coral sequences, and a subset of these validated by re-sequencing.

**Conclusion:**

The methods described here for deep sequencing of the transcriptome should be widely applicable to generate catalogs of genes and genetic markers in emerging model organisms. Our data provide the most comprehensive sequence resource currently available for reef-building corals, and include an extensive collection of potential genetic markers for association and population connectivity studies. The characterization of the larval transcriptome for this widely-studied coral will enable research into the biological processes underlying stress responses in corals and evolutionary adaptation to global climate change.

## Background

Reef-building corals support productive and diverse marine communities that provide important ecosystem services [1], but are threatened throughout their global range by a variety of stressors that include increasing sea surface temperatures, pollution, ocean acidification and disease [2-4]. A major focus of contemporary coral biology is to understand the factors determining stress resilience of corals and the potential for coral populations to recover from or adapt to these stressors [5]. The responses of coral holobionts to heat and light stress are especially complex because of the symbiotic association between reef-building corals and algae (zooxanthellae). The effects of light and temperature stress on these intracellular symbionts have been well characterized [6], but the genetic determinants of stress tolerance in the coral host, and any interaction effects between coral and zooxanthellae phenotypes, remain poorly understood [7,8]. Genomic resources such as genome or transcriptome sequences would make possible the detailed studies of gene expression, genetic connectivity, and stress physiology required for addressing these questions.

The genomic sequence resources currently available for corals are limited [9]. About 11,000 to 15,000 reads from pilot shotgun sequencing projects are available at NCBI for each of three corals (*Acropora millepora*, *Acropora palmata*, and *Porites lobata*), but no coral genomes have been completed and to our knowledge no genome sequencing projects are underway for any coral species. A number of studies have developed EST resources for corals using Sanger sequencing [10-12]. The staghorn coral *Acropora millepora *(Ehrenberg, 1834) has emerged as the most extensively sequenced scleractinian coral, with ~10,000 ESTs and ~14,000 shotgun genome sequences publicly available at NCBI. Additional EST sequencing projects using this species are ongoing but the results are not yet publicly available [13]. These EST sequencing efforts have allowed development of small-scale microarrays for gene expression analysis in the context of coral stress physiology [14], and similar studies aiming to identify stress candidates are currently underway [13]. These studies have highlighted the utility of cDNA sequencing for candidate gene discovery in the absence of a genome sequence, but a comprehensive description of the full complement of genes expressed in corals remains unavailable.

The increased throughput of next-generation sequencing technologies such as 454 sequencing [15] shows great potential for expanding sequence databases of corals and other emerging model organisms (reviewed in [16]). 454 sequencing of transcriptomes for organisms with completed genomes has confirmed that the relatively short (100–200 bp) reads produced by current versions of this technology can be effectively assembled and used for gene discovery [17,18]. These methods have not yet been widely applied to emerging model organisms, because of the lack of methods for *de novo *assembly and analysis in the absence of a reference genome sequence. Nevertheless, the few examples published to date have successfully demonstrated the potential for discovery of genes and genetic markers in these systems [19-23]. Despite their obvious potential, next-generation sequencing methods have not yet been applied to corals.

In this study, we describe improved methods for cDNA library preparation and titration for *de novo *transcriptome sequencing of any organism using 454, as well as sequence analysis and annotation procedures. The strategy outlined here does not require prior sequence knowledge, and relies exclusively on publicly available software and basic scripting tools. We applied these methods to sequence the larval transcriptome of the widely-studied coral *A. millepora*. The assembled, annotated sequences so produced provide a nearly complete catalog of the genes expressed in planulae larvae.

## Results

### 454 sequencing, assembly, and sequence analysis

**Figure 1:**
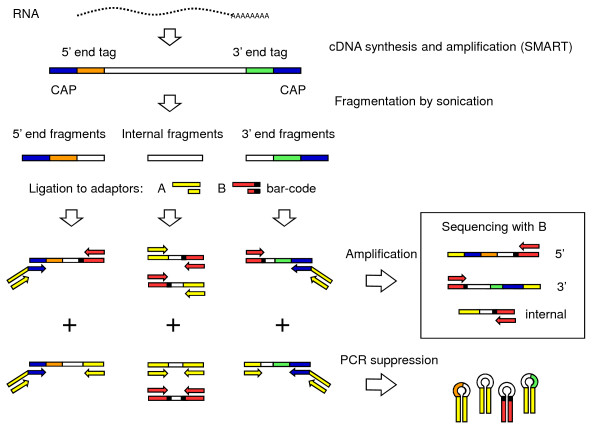
**Diagram of cDNA synthesis and 454 library preparation procedures**. Three fragment types are produced by sonication: 5', internal, and 3'. Ligation with the partially double-stranded adaptors A and B produces, for each fragment type, certain adaptor configurations that will be amplified (above the '+'), and others that will be suppressed (below the '+') during the subsequent amplification. The procedure preferentially amplifies constructs that are appropriate for 454 sequencing (shown inside box).

**Figure 2:**
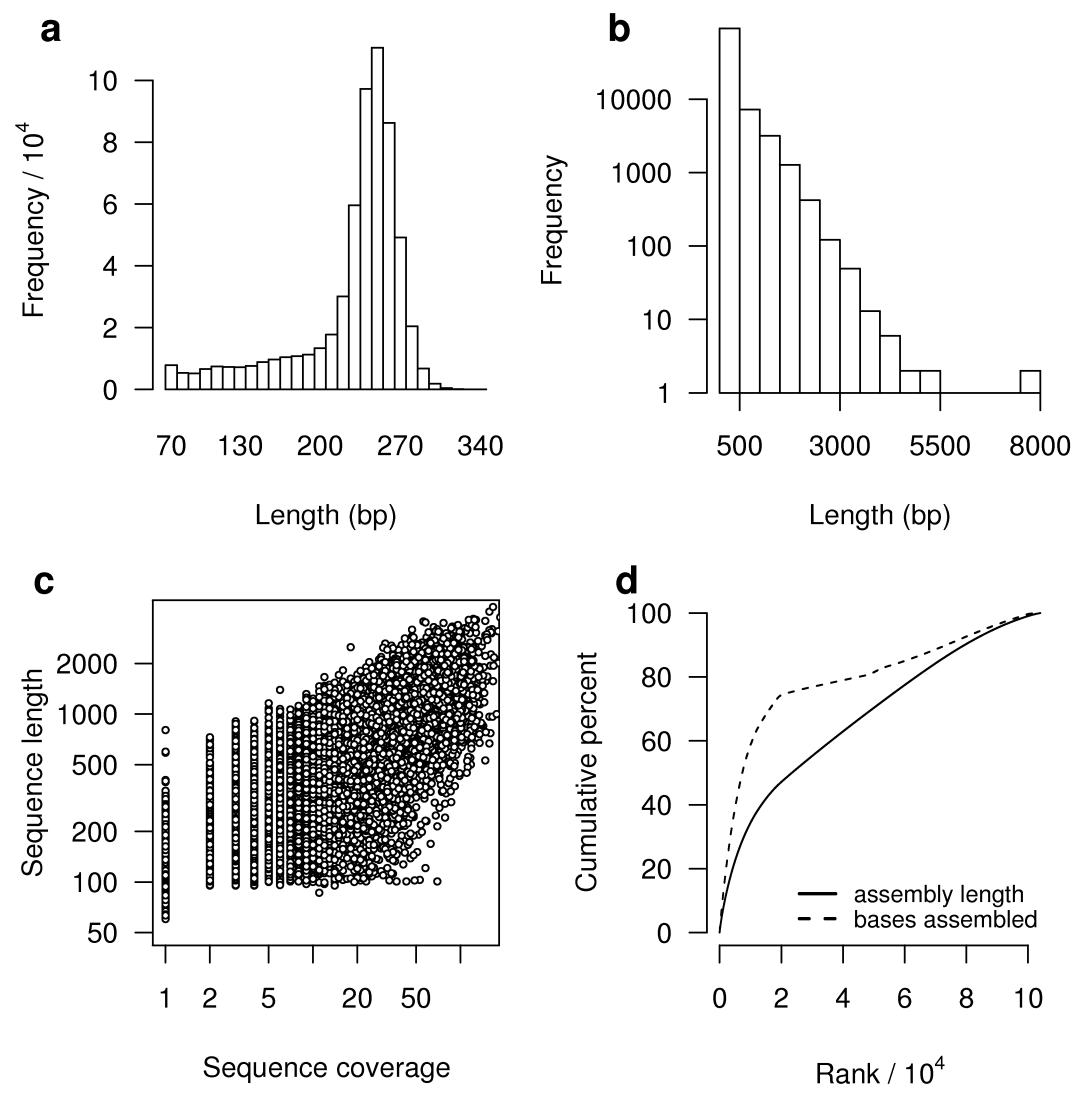
**Overview of *A. millepora *transcriptome sequencing and assembly**. (A) Size distribution of 454 sequencing read sizes after removal of adaptor sequences and outliers. (B) Size distribution of assembled sequences after assembly and contig joining. Note the logarithmic y-axis. (C) Log-log plot showing the dependence of assembled sequence lengths on the number of sequences assembled into each. (D) Assembled sequences are shown ranked from largest to smallest, with the cumulative percent of assembled bases (dashed line) and total assembly length (solid line) calculated based on those rankings. Sequence rank is shown in units of 10,000.

A cDNA sample was prepared from coral larvae as illustrated in Figure 1, and sequenced using the 454 GS-Flx platform. This single sequencing run produced 628,649 reads, with an average sequence length of 232 bases (SD = 55, range = 30–474). An overview of the sequencing and assembly is given in Table 1. After removal of adaptor sequences, 623,267 reads remained, with an average length of 216 ± 58 bases. This comparison revealed that 99% of the reads produced contained useful sequence data. A total of 146 Mb of raw sequence data were generated, of which 135 Mb remained after adaptor trimming (92% of sequenced bases). Size-selection to remove outlier reads (unusually long and unusually short) reduced this to 134 Mb (599,248 reads; 95% of the original number of reads). The size distribution for these trimmed, size-selected reads is shown in Fig 2A, revealing that 92% of trimmed reads fell between 100 and 300 bp in length.

**Table 1:** Summary of sequencing, assembly, and analysis

	Sequences (n)	Bases (Mb)
Raw sequencing reads	628,649	146.3
Trimmed & size-selected	599,248	133.6
Contigs	44,444	19.6
Singletons	62,657	13.6
Total	107,101	33.2
Scaffolds	104,005	
Sequence clusters	93,466	

Assembly of the trimmed, size selected reads along with the publicly available EST sequences (NCBI's dbEST) for *A. millepora *produced 44,444 contigs, with 62,657 reads remaining as singletons. Contigs ranged from 86 to 7,830 bp in size, with an average of 440 bp and an N50 of 693 bp (i.e., 50% of the assembled bases were incorporated into contigs 693 bp or longer). The size distribution for these contigs is shown in Fig 2B. The assembly produced a substantial number of large contigs: 11,813 contigs were >500 bp in length, and 4,654 were >1 kb. Sequencing coverage ranged from 1 to 65, with an average coverage of 5. As expected for a randomly fragmented transcriptome, there was a positive relationship between the length of a given contig and the number of reads assembled into that contig (Fig 2C). The majority of assembled reads were incorporated into the top 20,000 largest contigs, which included 74% of the bases sequenced and accounted for 47% of the total assembled length (Fig 2D).

Singleton sequences (i.e., reads that were not assembled into contigs) were retained on the basis that although some singletons might represent artifacts or contaminants, others are likely fragments of transcripts present at low levels in the original sample. This interpretation is supported both by analysis of sequence similarity and by PCR validation of singletons. 5,330 of the 62,657 singletons had significant blast matches in the Swiss-Prot database, and nearly half of these top hits (2,404) were not found among the top hits from contigs in this same database. This finding suggests that singletons contain sequence information not found among contigs, consistent with the possibility that they represent unique genes expressed at levels low enough to hinder adequate sampling. To confirm that these sequences were present in our experimental material (i.e., were not simply sequencing artifacts), we randomly selected 10 singletons for primer design. PCR analysis confirmed, for 8 of the 10 tested, that these sequences are present in the original cDNA (additional file 1). These 8 primer pairs each produced a single band of the expected molecular weight, and the others gave either no product or multiple bands. A large number of cycles (35–40) were required to produce detectable products, consistent with the possibility that these sequences represent genes expressed at low levels. Sanger sequencing of the eight successful PCR products confirmed the identity of five, with the other three matching poorly to the target singletons (Table 2).

**Table 2:** Validation of singleton sequences and the contig joining procedure by PCR amplification and Sanger sequencing.

Singleton ID	PCR	Aligned region (bp)	% identity
E60BDOM01CLWZX	+	127	95%
E60BDOM01ALGIK	+		
E60BDOM01BPLRZ	+	154	94%
E60BDOM02F1SCS	+	148	99%
E60BDOM01CV0LE	+		
E60BDOM01ETTGL	+	157	99%
E60BDOM01BOMBA	+		
E60BDOM01AM229			
E60BDOM01CMRH7			
E60BDOM01E1RA8	+	127	98%

Scaffold ID	PCR	5' match, bp (%identity)	3' match, bp (%identity)

EZ002257	+		193 (98%)
EZ000984	+	248 (99%)	123 (99%)
EZ001217	+	722 (96%)	374 (98%)
EZ001302	+	174 (97%)	180 (99%)
EZ001324	+	361 (97%)	183 (99%)
EZ002268	+	249 (99%)	186 (99%)
EZ001475	+		323 (99%)
EZ002219	+	715 (98%)	218 (98%)
EZ000750	+	768 (96%)	212 (99%)
EZ001662	+	133 (99%)	55 (98%)

Singleton sequences are identified by sequence identifiers for SRA003728, with the size (in bp) and quality (% identity) of the alignment between the 454 sequence and the Sanger sequence. Scaffolds are identified by accession numbers, with sequence identity shown for the 5'-most contig and the 3'-most contig contained in that scaffold.

Of the 107,101 assembled sequences (contigs plus singletons), 24,850 had significant matches in public protein sequence databases (nr). These blast matches correspond to 17,707 different unique accession numbers, of which 2,331 were each matched by multiple queries without overlap. These 2,331 subject sequences correspond to 5,327 different query sequences (~2.3 queries matched each subject, on average). For each of these protein sequences, all queries matching that subject were joined together in the appropriate order and strand (sense/antisense) to produce a scaffold, a construct intended to represent the collection of fragments originating from a single transcript. Overall, the contig joining process condensed the number of sequences from 107,101 to 104,005. To validate the contig joining procedure, ten sets of sequences that had been joined in this fashion (i.e., 10 scaffolds) were randomly selected for validation. Note that because the gaps in scaffolds are of unknown length, the scaffold sequences provide minimum expectations for PCR product size. All ten scaffolds were successfully amplified by PCR (additional file 1). Sanger sequencing of these PCR products confirmed the identity of eight scaffolds at both ends, and the other two at only one end (Table 2). These findings confirm that the scaffolds produced from our contig joining procedure successfully recapitulate cDNA sequences in most cases. This strategy successfully identified different parts of the same transcript for >2,000 transcripts in our study, and should be widely applicable for other transcriptome sequencing efforts in emerging model organisms.

The preceding steps, including assembly of raw reads and contig joining, were expected to reduce redundancy among sequences (i.e., more than one sequence per gene). To characterize any redundancy that remained, assembled sequences were clustered using two parallel procedures based on (1) nucleotide sequence similarity among the assembled sequences themselves, and (2) protein sequence similarity among the query sequences' best blast matches. The final set of 93,466 merged clusters was obtained from the union of these protein and nucleotide sequence clusters. Excluding the large clusters matching transposable elements, sequence clusters ranged from 1 to 64 sequences in size, with most containing only 1–2 sequences and a small number of clusters containing more than 2. The largest clusters could indicate paralogs, highly-divergent alleles, well-conserved gene families, alternative splice variants, or simply high levels of expression with correspondingly high sequencing error due to increased sampling. Regardless of the biological interpretation of sequence clusters, the clustering of 104,005 assembled sequences into 93,466 clusters indicates that relatively little redundancy remained after assembly and joining. Annotation of sequences clusters allows the rapid identification of all sequences with similarity to a particular gene of interest, facilitating studies of sequence polymorphisms and closely related genes.

### Functional annotation of the transcriptome

Because the significance of sequence similarity depends in part on the length of the query sequence, short sequencing reads obtained from next-generation sequencing (e.g., the ~216 bp average trimmed read length obtained in this study) frequently cannot be matched to known genes [21]. Most of the 104,005 assembled sequences analyzed here are short (61% ≤ 250 bp), and correspondingly few have significant blast matches in NCBI's nr database (23.9%). The proportion of sequences with matches in public databases is greater among the longer assembled sequences, as demonstrated by applying a series of increasing minimum size cutoffs and recording the number of queries with significant matches (additional file 2). Among sequences ≥ 300 bp (i.e., longer than a single read), significant matches were found in nr for 62%, and among those longer than 1 kb, the proportion increased to 88.8% (Table 3).

**Table 3:** Summary of annotation of the A. millepora larval transcriptome.

	All sequences	≥ 300 bp	≥ 1000 bp
Total number of sequences	104,005	19,210	5,039
Sequences with BLAST matches	24,850	11,901	4,474
Sequences matching known genes	15,860	9,464	3,889
Sequences assigned GO terms	17,902	8,915	3,436
Sequences with conserved domains	12,785	8,144	3,573

Assembled sequences were assigned gene names based on the gene product and gene name annotation of the best blast match for that sequence. This procedure successfully assigned gene names for 15,860 sequences among the entire dataset; for 9,464 of the sequences ≥ 300 bp in length; and for 3,889 of the sequences ≥ 1 kb in length (Table 3). Among the 15,860 annotated best hits, 11,633 different gene names were assigned, providing a rough estimate of the number of different genes expressed in these libraries. This is probably an underestimate because many sequences lacked matches in public sequence databases (Table 3) and were therefore not assigned gene names.

Analysis of protein domains revealed that 12,785 of the assembled sequences matched profiles in NCBI's conserved domains database, corresponding to 4,475 different domains. Most domains were found in only 1–2 sequences, with a small number appearing more frequently. The top 20 most frequently detected domains include conserved domains associated with transcription factors, growth factors, and signaling pathways. The utility of domain annotation is that it allows researchers to quickly select all genes sharing a common domain; for example, selecting the 60 different sequences matching the domain COG5576 (homeodomain) retrieves putative homologs of 51 different genes with known roles in development and morphogenesis (e.g., *SIX4*, *PAX3*, *dll*, and *engrailed*).

Gene Ontology terms were assigned to 17,902 assembled sequences based on sequence similarity with known proteins annotated with GO terms (UniProt-TrEMBL database). For each sequence, the specific annotated terms were mapped to higher-level (i.e., more general) parent terms to provide a broad overview of the groups of genes cataloged in this transcriptome for each of the three ontology vocabularies. The hierarchical structure of these vocabularies allows the selection of sets of genes involved in a specific process at the desired level of detail. For example, GO annotation revealed 320 sequences implicated in general stress responses, including heat shock factor binding proteins (Table 4). Within this set of general stress response genes, subsets of genes were associated with specific stressors (i.e., higher level GO terms), including heat stress (e.g., heat shock protein 70), oxidative stress (e.g., catalase), and response to wounding (e.g., phospholipase A2-activating protein). These searches were based on processes that are the focus of ongoing research in coral biology, and identified a number of genes that had not previously been investigated in the context of coral stress responses (Table 4). These GO annotations provide a valuable new resource for investigation of specific processes, functions, or cellular structures involved in coral stress responses.

**Table 4:** Candidate genes identified based on GO annotation of *A. millepora *larval transcriptome.

Process	GO Term	Sequences	Example gene (match accession)
Response to stress	0006950	320	Heat shock factor-binding protein (Q5RDI2)
Response to heat	0009408	2	70 kDa Heat shock protein (P17879)
Response to oxidative stress	0006979	42	Catalase (Q9PWF7)
Response to wounding	0009611	4	Phospholipase A2-activating protein (P27612)
Apoptosis	0006915	38	Caspase (P70677)
Exocytosis	0006887	7	Exocyst complex component 5 (P97878)
Immune response	0006955	58	H-2 class II histocompatibility antigen (P04441)
Nitric oxide metabolism	0046209	3	Nitric oxide synthase (O19132)
Protein folding	0006457	123	60 kDa heat shock protein (P18687)
Vacuolar transport and organization	0007034, 0007033	5	Vacuolar protein sorting-associated protein 26B-B (Q6DH23)

### Pathways and complexes

To evaluate the completeness of our transcriptome library and the effectiveness of our annotation procedure, we searched the annotated sequences for the genes involved in a set of metabolic pathways and protein complexes shared among animal phyla. These simple text searches were based on standard gene names or synonyms. This confirmed that our data include annotated sequences for all genes in the five major pathways considered here (Table 5). In a similar set of searches for components of essential protein complexes, almost all the genes were found (91–100%; Table 5). The presence of these essential cellular process genes suggests that these annotated sequences accounts for nearly the complete coral larval transcriptome.

**Table 5:** Genes from essential metabolic pathways and macromolecular complexes annotated in larval transcriptome.

Target	Genes found (n)	Known genes (n)
Pathways		
Glycolysis	10	10
Gluconeogenesis	10	10
Pentose phosphate	5	5
Citrate cycle	9	9
Urea cycle	5	5
Complexes		
26S proteosome	22	22
Chaperonin (TCP1)	8	8
Spliceosome	130	143
Ribosome	76	79
Nuclear pore complex	26	28

To further evaluate the depth of coverage in this library, we searched the annotated sequences for sets of regulatory genes known to be present in anemone *Nematostella vectensis*, the most closely related organism for which an assembled draft of the genome sequence is available. The rationale for these searches was that the presence of these genes in anemone suggests their presence in coral, and their successfully identification in the coral larval transcriptome would support the completeness of this sequencing effort. For a set of 24 genes associated with eight major intercellular signaling pathways, 23 were successfully identified in the transcriptome sequences (Table 6). Activin-like kinase was notably absent from this list, but this finding does not imply the absence of this gene in *A. millepora*; it could simply indicate that the gene is not expressed, or expressed at a very low level, in the developmental stages sampled in this study.

**Table 6:** Intercellular signaling pathway genes annotated in larval transcriptome.

Pathway	Gene name	Sequences (n)
Hedgehog	Patched	15
	Fused	1
	Hedgehog	6
	Smoothened	2
JAK/STAT	Janus kinase	4
	Signal transducer and activator of transcription	1
NFKB/Toll	Nuclear factor NF-kappa-B	3
	Toll-interacting protein	1
	Toll-like receptor	9
NHR	Estrogen-related receptor	1
	Hepatocyte nuclear factor 4	1
	Retinoid-related orphan receptors	6
Notch	Furin	8
	Delta	12
	Notch	23
	Presenilin	4
	TACE	1
RTK	Receptor tyrosine kinase	5
TGF-beta	Activin-like kinase	0
	SMAD	15
	TGF-beta-receptor	3
WNT	Disheveled	2
	Frizzled	22
	Wnt	25

A similar search was conducted for a set of transcription factors known to be present in *N. vectensis*, based on conserved domains characteristic of each transcription factor family. Of the 20 families investigated, these searches identified annotated sequences corresponding to 18 (Table 7). These text searches were based on domain identifiers, domain descriptions, and gene names. No matches were found for two families of transcription factors: the cold-shock DNA binding domain (pfam00313), and the paired amphipathic helix repeat (pfam02671). The lack of detection of these genes is not evidence that they are absent in the coral larvae; it might indicate their low expression, incomplete sequencing coverage, or inadequate annotation. Nevertheless, the overall success in identifying the known sets of genes associated with these pathways and complexes indicates that the collection of sequences established in this study provides a reasonably complete description of the coral larval transcriptome.

**Table 7:** Major transcription factor families identified by conserved domain annotation of larval transcriptome

Sequences (n)	Domain ID	Conserved domain description
1	pfam01722	BolA-like protein
0	pfam00313	Cold-shock DNA-binding domain
1	pfam01381	Helix-turn-helix
2	pfam02229	Transcriptional Coactivator p15
0	pfam02671	Paired amphipathic helix repeat
1	pfam02864	Signal transducer and activator of transcription
1	pfam01167	Tub family
4	pfam00046	Homeobox domain
1	pfam00447	HSF-type DNA-binding
2	pfam00870	P53 DNA-binding domain
6	pfam02257	RFX DNA-binding domain
2	pfam01422	NF-X1-type zinc finger protein
3	pfam02319	E2F/DP winged-helix DNA-binding domain
1	pfam00319	SRF-type transcription factor
1	pfam00250	Fork head
12	pfam07716, pfam00170	Basic region leucine zipper & bZIP
9	pfam00010	Helix-loop-helix DNA-binding domain
3	pfam00249	Myb-like DNA-binding domain
12	pfam00642	Zinc finger, CCCH type
5	pfam00096	Zinc finger, C2H2 type

### Comparisons with *Nematostella vectensis*

Sequence comparisons revealed broad similarity between the coral sequences described here and the protein sequences predicted from the anemone genome. Of the 104,005 assembled coral sequences, 20,619 have significant matches among the anemone predicted proteins (blastx with adjusted e-value threshold of 10^-4^). These correspond to 11,572 different predicted proteins. Reciprocal blast searches, in which the anemone sequences were queried against the coral sequences, revealed that 20,976 of the 27,273 *N. vectensis *proteins had significant similarity with coral sequences (tblastn), corresponding to 9,802 different coral sequences. Comparison of the best blast matches from these searches identified 8,515 unambiguous orthologs between coral and anemone, based on significant reciprocal best matches. Because the coral sequences represent partial transcripts, this is likely an underestimate of the true number of anemone orthologs expressed in this developmental stage.

To investigate whether the coral larval transcriptome contains "coral-specific" genes not found in the anemone, the assembled coral sequences were first compared with public databases. 22,158 coral sequences had significant matches in nr with an e-value ≤ 10^-4^; these represent sequences with reasonably strong matches to previously identified proteins in other organisms. A list of all coral sequences that showed even weak similarity to anemone proteins was compiled by searching the predicted proteins (blastx) and the genome assembly (tblastx), using a permissive threshold of e ≤ 0.01 (n = 28,190 sequences). Comparison of these results revealed that 748 coral sequences matched known genes in other organisms but had no significant similarity to anemone proteins from the draft genome. To reduce false positives stemming from the fact that many assembled sequences were not complete cDNAs, the top blast match for each of these (i.e., a complete protein sequence for that gene, from a different organism) was queried against the anemone proteins with a permissive threshold of e ≤ 0.01. After eliminating the sequences with matches in this search, 207 sequences remained, corresponding to 95 different annotated coral genes. These analyses suggest that these coral genes lack orthologs in the anemone. Because the draft assembly of the anemone genome is not complete [24], further experimental work would be required to verify the absence of these candidate coral-specific genes in the anemone.

### SNP detection and validation

**Figure 3:**
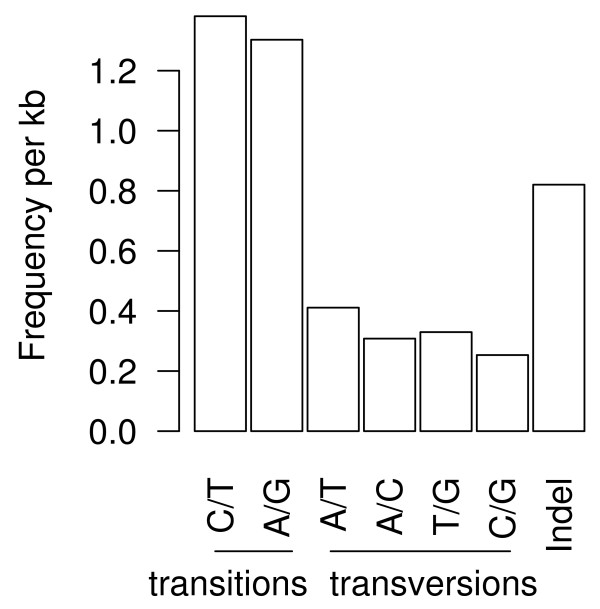
**Classification of single nucleotide polymorphisms (SNPs) identified from 454 sequences**. Overall frequency of these SNP types in the larval transcriptome is one per 207 bp.

Using the QualitySNP program, we identified 33,433 high quality SNPs and 6,820 indels within 14,613 CAP3-assembled contigs (Fig 3). These predicted SNPs included 22,312 transitions and 10,823 transversions. The overall frequency of all SNP types in the transcriptome, including indels, was 1 per 207 bp. These predictions provide an extensive set of genetic markers for reef-building corals that will enable future studies of genetic connectivity and genetic mapping at a previously unprecedented level of detail.

Twenty of these predicted SNPs (corresponding to 18 different genes) were selected for validation using PCR and Sanger sequencing, and 14 of these tests (70%) were successful (additional file 3). Because the genes selected for validation include several previously described stress responses genes [14], the SNPs confirmed in these analyses may be valuable for investigating the genetic determinants of stress-gene expression. Overall, the successful experimental validation of the majority of computationally predicted SNPs confirms the utility of mining 454 transcriptome sequences for genetic markers.

## Discussion

### Methodological advances for 454 sequencing of transcriptomes

The methods described here resolve several challenges associated with 454 sequencing of transcriptomes. Because 454 sequencing does not efficiently process homopolymer regions greater than 8 bp in length [15], the poly-A tails at the 3' ends of intact transcripts present a problem, and would be expected to result in under-representation of the 3' ends of transcripts. In our method, this is resolved by simply interrupting the poly-T region with a single C. A single copy of this 'broken-T' adaptor was found in 98,276 of the raw reads (16% of the total), slightly more than the expectation (~10%) based on average read length (232 bp) and assuming an average transcript size of 2,200 bp. This comparison suggests that the 3' ends of transcripts were well-represented in our dataset, confirming the effectiveness of this solution for overcoming the homopolymer problem. Another difficulty in 454 transcriptome sequencing is concatenation of adaptors during cDNA preparation, an issue that is commonly encountered but seldom reported because concatenated adaptor fragments either fail to sequence or are removed by adaptor trimming, decreasing the final sequencing yield. Our library preparation method resolves this difficulty by (1) sequencing all fragments from an internal end, so that any concatenated adaptors encountered will tend to be found at the end of the read; and (2) avoiding the over-amplification that can contribute to adaptor concatenation. The emPCR titration method described in this study reflects an additional improvement on previous methods that further increases the number of usable reads obtained per run.

Comparison of our results with recently published studies that employed 454 sequencing confirms the effectiveness of these improvements. First, 95% of the reads produced in our study passed stringent quality filters (i.e., adaptor trimming and size selection), an improvement over the 85–90% that passed similar quality filters in previous studies [17,18,23]. Our assembly incorporated 90% of high-quality reads, improving on the 40–88% reported for assemblies in previous studies [17,21,23]. The average contig size, which is strongly affected by read length, was ~90 bp higher in our study than in a recently published study that also used the 454 GS-Flx sequencing platform and so began with reads of comparable length [21]. Our assembly also included a larger proportion of long contigs (27% of contigs were ≥ 500 bp) than the 8% reported in [21], despite the larger number of reads (>1 million) from multiple sequencing runs in that study. Finally, our library preparation and titration procedures produce 2–5 times more reads per sequencing run than standard methods: ~620,000 in our study versus ~130,000 to ~390,000 in previous reports [17,21-23]. One possible caveat for these comparisons is that our assembly did include a small number of Sanger ESTs (~11,000), and these might be expected to improve the assembly. However, the effect of these Sanger ESTs on the assembly was actually negligible: a separate assembly that excluded those ESTs produced only 0.4% more contigs, 1% more scaffolds, and 4.8% shorter contigs. Overall, these comparisons with previous studies highlight the potential for our method to improve sequencing depth and efficiency in future transcriptome sequencing efforts.

Deep sequencing of transcriptomes with 454 sequencing typically produces many singleton sequences that fail to assemble, representing 10% of the reads in our study and 13–60% in other recent 454 transcriptome assemblies [17,19,23]. Singletons could result from sequencing errors, artifacts of cDNA normalization, genes expressed at low levels in the coral, or contaminants from other sources. For example, our cDNA was derived from larvae grown in non-sterile conditions and exposed to crustose coralline algae (CCA) collected from the reef, suggesting potential sources of contamination. To test for differences between cDNAs that remained as singletons and those that were assembled into contigs, a subset of 10,000 reads was randomly selected from each set. Because this comparison was based on sequencing reads, which did not differ in size between the two sets, this comparison avoided the confounding effect of sequence length. Contig reads and singleton reads were compared separately against nr using blastn (e-value ≤ 10^-6^), and the taxonomic origin of their top blast matches was recorded. This confirmed that most sequences in both sets best matched metazoan sequence records from other species, although singleton sequences contained a significantly lower proportion of metazoan matches (89%) than contig reads (95%) (Fisher's exact test; *P *< 0.01). Singletons contained a significantly higher proportion of sequences that best matched proteobacteria (*P *< 0.01) and amoebozoa (*P *< 0.05) records than reads that assembled into contigs, suggesting that singletons were enriched in cDNA from biological contaminants. However, the absolute numbers of putative contaminant hits were still very low (3–4 sequences for each non-metazoan group), suggesting that the contamination level was low overall. A notable difference revealed by these comparisons is that a larger proportion of contig reads (10%) had significant matches than singleton reads (2%) (Fisher's exact test; *P *< 0.001). This suggests that a substantial fraction of singletons (but not all) were artifacts of cDNA synthesis, normalization, or sequencing. Despite the potential artifacts and contaminants detected among singletons, 5171 of the total (62,657) sequences matched annotated protein sequences in the nr database, corresponding to 3989 different genes (blastx, e-value ≤ 10^-4^). These findings support the inclusion of singletons in our analysis on the basis that many represent coding sequence and so could be biologically interesting despite their low expression.

### Estimating the number of genes expressed in larvae

One of the primary questions for transcriptome sequencing projects is the number of genes expressed, but in organisms lacking a fully-sequenced genome, addressing this question is hampered by several unknowns. First, some contigs are expected to lack matches in public databases and therefore remain as separate sequences after contig-joining. Other genes present in a newly-sequenced transcriptome might be represented by fragments that match conserved regions in known genes, as well as fragments corresponding to poorly-conserved regions (e.g., un-translated regions) that would be expected to lack matches. Finally, some sequence fragments might be too short to allow for statistically meaningful matches, regardless of which transcript they were derived from. At the level of coverage obtained in a single sequencing run (~5-fold), sequence assembly alone is not sufficient to produce complete transcript sequences. Because the contig joining procedure described here relies on protein sequences similarity, it therefore cannot act on sequences without protein matches. No obvious solutions are available for assembling the sequences from a non-model system transcriptome sequencing project into a number of contigs that precisely matches the number of genes expressed, although paired-end library sequencing might improve the situation.

To arrive at an estimate of the number of genes expressed in this library, several alternative strategies are available that take into account the sources of uncertainty described above. (1) Gene number can be estimated based on the number of well-supported, unique sequences in the assembly as follows. The majority of sequenced bases (>70%) assembled into sequences at least 300 bp in length (Fig 2D), excluding essentially all singleton sequences (Fig 2A). The accuracy of these 19,210 assembled sequences is well supported by their high sequencing coverage (24 reads per assembled sequence, on average). Among these high-quality sequences, 16,360 sequence clusters (representing groups of highly similar sequences) were identified, providing one estimate of the number of genes expressed in these libraries. (2) Alternatively, gene number can be estimated based on the assembled sequences that matched known genes, as follows. Among those contigs sufficiently long to allow good matches (≥ 300 bp), 11,901 showed significant similarity to records in the nr protein database. Gene names were assigned to 9,464 of these on the basis that they matched known genes with annotated coding sequences, corresponding to a non-redundant list of 7,815 different gene names. Of course, many cnidarian genes are expected to lack matches in public databases; for example, 19% of predicted proteins from the anemone *N. vectensis *lack matches to other known genes [25]. Based on the assumption that a similar proportion of coral genes would lack matches to known genes, this suggests an adjusted estimate of 9,591 genes expressed in *A. millepora *larvae (i.e., 7,815/0.81). (3) Finally, gene number can be estimated based on comparisons with the anemone *N. vectensis*. Based on blastx comparisons, 20,617 of the assembled coral sequences matched to 11,572 different predicted proteins from the anemone genome. Because more redundancy and gaps are expected among our assembled coral sequences than in the draft anemone genome, this should approximate the number of genes expressed in our libraries. Although no clear solutions are available to conclusively determine gene numbers without a fully-sequenced genome, the three estimates described suggest that ~11,000 different genes are expressed in larvae of *A. millepora*.

This estimate matches well with previous estimates of the number of genes expressed during metazoan development. About 11–12,000 genes are expressed during sea urchin development based on whole-genome tiling arrays [26]. At least 6000–8000 genes are expressed during each embryonic or larval stage of nematode development, based on SAGE libraries [27]. About 10,000 genes are expressed in fruit fly embryos [28]. Comparison with these previous studies that span different animal phyla and analytical techniques reveals that our estimate of 11,000 genes expressed in coral larvae is within the expected range for metazoan development.

### Sequencing depth and annotation success

To evaluate whether the sequences annotated in this study includes all genes expressed in these treatments and developmental stages, we searched for a number of genes involved in metabolic pathways and macromolecular complexes involved in essential cellular processes. For these searches, the lists of genes associated with metabolic pathways were based on the BioCyc database of pathways [29], and gene lists for protein complexes were based on the CORUM database of mammalian protein complexes [30]. The rationale for these searches was that since those essential genes must be expressed to maintain cellular functions, any failure to find these sequences in the transcriptome would reflect either inadequate sequencing depth or ineffective annotation. For the pathways and complexes considered here, essentially all genes (91–100%) were found based on these simple text searches (Table 5). A caveat for these findings is that high levels of expression might be expected for of some essential 'housekeeping' genes, causing them to be well represented in even an incomplete transcriptome sequencing effort. To account for this possibility, we also searched for genes associated with intercellular signaling pathways and transcription factor genes. Because of their more restricted spatial and temporal patterns of expression, these genes are not expected to be as highly expressed in whole-organism libraries as the essential 'housekeeping' genes. The query lists for these searches were based on genes and pathways conserved among primitive metazoans and cnidarians [12,25,31]. These searches successfully identified genes from nearly all of the pathways and transcription factor families considered (Tables 6, 7). The few genes that could not be found might result from incomplete annotation, inadequate sampling of the transcriptome, or genes that are truly not expressed. Regardless of the cause, the impact appears to be minimal (i.e., almost all genes from these pathways were found). Overall, these searches support the conclusion that the collection of annotated sequences produced in this study represents a reasonably complete description of the coral larval transcriptome.

### Applications for measuring gene expression

One immediate application for the annotated sequence resource developed in this study is the design of microarray probes for gene expression analysis of biological processes such as heat tolerance or innate immunity. To that end, we have selected a set of 11,000 sequences for probe design based on the following criteria. To ensure that all sequences were sufficiently long to allow design of high-quality probes, we selected the subset of sequences ≥ 250 bp in length (n = 40,686). Among these, 10,652 matched annotated genes in public sequence databases, allowing unambiguous identification of the sense (protein-coding) strand. These included 8,523 clusters of similar sequences; within each cluster, sequences were ranked by coverage, and the most highly-covered member of each cluster was selected. Finally, based on gene name annotation, for any cases where two sequences were annotated with the same gene name, the sequence with higher coverage was selected. This produced a set of 7,930 well-annotated sequences corresponding to known genes. These sequences have been adjusted to ensure that the sense strand is represented, based on blast matches. To bring the total to 11,000, the remaining sequences ≥ 250 bp in length without matches in public databases (n = 30,034) were ranked by coverage and the 3,070 most highly covered sequences selected. For those sequences without blast matches, the strand information (sense/antisense) is not known. One important practical note about the use of these sequences for gene expression analysis is that probes or primers designed based on these sequences should be designed so as not to span the junctions produced by contig-joining. The set of sequences selected above provides a valuable resource for designing microarray probes, and the current widespread use of microarrays for gene expression profiling of corals [32,33] suggests that this resource will be immediately useful.

### Development of genetic markers

Although *A. millepora *is an emerging coral molecular biology model for molecular ecology studies, few genetic markers are currently available for this species [34]. These markers are obviously not enough for genome-wide association studies of coral physiological variation, which is the focus of ongoing projects in our laboratory addressing the molecular and evolutionary mechanisms of coral adaptation to climate change. This study, like others recent published [23,35], shows a cost-effective way to produce a large number of gene-associated SNPs from transcriptome data obtained by 454 sequencing. QualitySNP is a newly developed program and uses a haplotype-based strategy to detect reliable SNPs without requiring sequence traces, quality scores, or genomic sequence data [36]. In our study, 70% of predicted SNPs were confirmed experimentally. These findings clearly demonstrate the utility of SNP mining from 454 transcriptome sequences, and provide the most extensive genetic marker resource currently available for *A. millepora*.

## Conclusion

In this study, we describe methods that will facilitate transcriptome sequencing for organisms that present important biological questions but lack fully-sequenced genomes. The laboratory procedures outlined here provide a rapid and cost-effective method for deep transcriptome sequencing in a single 454 sequencing run, improving substantially on previous methods. The analytical strategies we describe for adaptor trimming, assembly, joining, and annotation can all be accomplished using publicly available programs and basic scripting tools, and produce useful collections of annotated sequences from short reads. Our findings provide a nearly complete description of the genes expressed in coral larvae, a resource that is expected to be immediately useful for measurements of gene expression in reef-building corals, in addition to a large number of genetic markers for studies of genetic connectivity and structure. The single 454 sequencing run employed in this study produced more than 600,000 sequencing reads, allowing the identification of ~11,000 genes and ~30,000 SNPs. These findings represent a substantial contribution to the existing sequence resources for reef-building corals. Application of these resources will greatly enhance our understanding of the potential for corals to adapt to increasing environmental stress during climate change.

## Methods

### Larval culture and RNA extraction

Because adult coral colonies contain algal symbionts that could result in contamination of extracted RNA, our experiments focused on a developmental stage (planulae larvae) that, in this species, lacks those symbionts. Adult colonies of *Acropora millepora *were collected at Magnetic Island (Townsville, QLD, Australia) and placed separately into individual bins prior to spawning. Corals were allowed to spawn naturally, following the synchronized mass spawning schedule typical for this and many other coral species [37], and then returned to their original locations on the reef. For each colony, gamete bundles were gently sieved through a 130-μm pore-size nylon mesh to separate sperm from eggs. Eggs from one individual colony were suspended in 1 l of 1-μm filtered seawater (FSW), and fertilized with sperm from a different individual colony. After allowing ~5 h for fertilization, excess sperm was removed by rinsing with FSW and embryos were stocked at 2 per ml in 2-l plastic culture vessels filled with FSW.

To maximize the diversity of expressed genes in our experimental material, we exposed these larvae to a range of different treatments expected to affect gene expression. These treatments included temperature stress and known inducers of settlement and metamorphosis, in order to induce expression of the genes associated with those processes. Three replicate cultures were maintained for 5 d at a standard culturing temperature of ~28°C, and another three replicates at an elevated temperature of ~32°C. Culture water was exchange for fresh FSW regularly throughout development.

Larvae grown at the standard (lower) temperature were pooled between culture replicates at 5 d post-fertilization and those pooled larvae used in short-term treatments expected to affect gene expression. One group was incubated at the higher temperature for 4 h (heat stress). Another group was not given any additional treatment, to serve as a control for any effects of handling during these short-term treatments. A third set of larvae from these same cultures was incubated with a known natural inducer of larval settlement [38]: crustose coralline algae (CCA). Other larvae were incubated with an artificial inducer of metamorphosis, a GLW-amide peptide called EPLPIGLW-amide [39]. Using light microscopy, we confirmed that the CCA treatment induced behavioral and morphological changes associated with settlement, and the GLW-amide treatment induced metamorphosis directly, bypassing those subtle changes. After 4 h incubations in each treatment, the larvae or recruits were preserved in RNALater (Ambion, Austin, TX, USA). Larvae were sampled from the different long-term culture conditions (28 and 32°C) on that same day and stored in the same manner, for a total of six different samples. RNA was extracted from these samples using the Ambion RNAqueous-micro kit (Ambion) according to the manufacturer's instructions.

### Preparation of cDNA samples

The methods developed in this study for cDNA synthesis, amplification, and fragment library preparation in preparation for 454 sequencing are illustrated in Fig 1. RNA preparations were purified by precipitation with lithium chloride prior to cDNA synthesis. First-strand cDNA was produced using this purified RNA according to Clontech's SMART cDNA synthesis kit, with the following modifications. The primer used for first strand synthesis was a modified oligo-dT primer (AAGCAGTGGTATCAACGCAGAGTCGCAGTCGGTACTTTTTTCTTTTTTV). The poly-T stretch is broken by the inclusion of an internal C to minimize the potential for 454 sequencing problems in this homopolymer stretch. The remainder of cDNA synthesis protocol was conducted according to the manufacturer's instructions.

cDNA was amplified using the "CAP" primer (AAGCAGTGGTATCAACGCAGAGT) according to the manufacturer's instructions (SMART cDNA synthesis, Clontech), taking advantage of the PCR Suppression effect to preferentially amplify longer molecules and thus enrich for full-length transcripts [40]. Throughout these cDNA amplification procedures, precautions were taken to minimize distortions of the samples based on previously described principles [41]. Following amplification, PCR products were evaluated by gel electrophoresis to confirm that the appropriate range of molecular weights had been produced (0.5–3 kb), and that the libraries were not over-amplified (i.e., more than 200 ng PCR product per 30-μl reaction), which can distort expression profiles and lead to adaptor concatenation. To maximize the amount of fully double-stranded PCR product, the reactions were 'chased' with additional cDNA amplification primer (1 μl of 10 μM stock) and incubated at 78°C for 1 min, 65°C for 1 min, then 68°C for 3.5 min. Multiple PCR reactions were conducted for each library, then pooled and purified using the Qiaquick PCR Purification kit (Qiagen, CA, USA). Finally, equal quantities of cDNA from each of the six libraries were pooled to produce a library expected to include all genes expressed in the different treatments.

To minimize differences among the abundance of different transcripts (i.e., of genes expressed at different levels), 2 μg of the pooled, amplified cDNA was normalized using the Trimmer kit (Evrogen, Moscow, Russia) according to the manufacturer's instructions. This sample contained equal amounts of cDNA from each library (i.e., two different long term culture conditions and four different short term treatments). Following normalization, the cDNA was amplified for 15 cycles as described above. 16 reactions (30 μl each) were amplified, then pooled and purified as described above (Qiaquick PCR purification).

cDNA was sheared by sonication to produce short, random fragments appropriate for 454 sequencing. This was accomplished by first preparing 5 μg of amplified, normalized cDNA in a 100-μl volume. The pooled sample was submerged in an icewater bath and sonicated using a Misonix S3000 (Misonix, NY, USA) in 30 sec bursts followed by 30 sec rests, with power set at 0.5–1 (18–30 W). Fragmented cDNA samples were evaluated by gel electrophoresis to visualize the effectiveness of the process. A range of different sonication durations were tested, and the 3 minute treatment selected because it produced fragments in the appropriate size range (ca. 300–400 bp).

Oligonucleotide adaptors were ligated to the fragmented cDNA to facilitate 454 sequencing. First, the fragmented cDNA was 'polished' using Klenow and T4 DNA polymerases (New England BioLabs, Ipswich, MA, USA) to fill in single-strand overhangs that might remain after sonication. A partially double-stranded adaptor containing the standard 454 sequencing primer 'B' was prepared by combining oligonucleotides 'B+1' (GCCTTGCCAGCCCGCTCAGACGAGCGGCCA) and its partial complement, 'anti-B+1' (TGGCCGCTCGT) at a final concentration of 10 μM each. A second partially double-stranded adaptor containing the standard 'A' sequence was prepared by combining oligonucleotides 'A+' (GCCTCCCTCGCGCCATCAGCCGCGCAGGT) and 'anti-A+' (ACCTGCGCGG). The partially double-stranded structure of these adaptors ensures that they are ligated in the correct orientation and minimizes tandem ligations, since T4 DNA ligase requires a double-stranded DNA template. Adaptors were ligated to fragmented cDNA using T4 DNA ligase (Promega, WI, USA) in an overnight reaction at 12°C. Following ligation, constructs were purified using the Qiaquick PCR Purification kit to remove excess adaptors and reagents (Qiagen, CA, USA).

Ligations were tested by PCR amplification using different combinations of primers to verify that the correct constructs had been produced. Constructs were then amplified through a 'step-out' PCR scheme, using primers 'A' (GCCTCCCTCGCGCCATCAG) and 'B' (GCCTTGCCAGCCCGCTCAG), each at 0.2 μM final concentration, and 'A+CAP' (GCCTCCCTCGCGCCATCAGCCGCGCAGGTAAGCAGTGGTATCAACGCAGAGT) at 0.01 μM. Amplification was carried out for 17 cycles as described above, including chasing with an additional aliquot of primers and column purification following PCR. Finally, the constructs were labeled with biotin by repeating the amplification using a 5'-biotin-labeled 'A' primer. For this final labeling reaction, primers 'biotin-A' and 'B' were each used at 0.2 μM, and the reactions were amplified for 3 cycles. The final product was again column purified, to remove unincorporated oligonucleotides and biotin, and stored at -20°C prior to sequencing. Note that because our library preparation method explicitly controls for adaptor structure (i.e., only the correct constructs are amplified), the biotin labeling steps, which are required in typical 454 library protocols, are not actually required for our method.

A step-by-step protocol for the library preparation method outlined above is provided in the additional files accompanying this article (additional file 4). That protocol includes minor modifications that we have recently developed to reduce the number of manipulations required, improve reproducibility, and to further reduce adaptor concatenation. The improved protocol in that file is recommended for researchers interested in using our methods for 454 sequencing of transcriptomes.

### Library titration and 454 sequencing

The biotin-labeled library described above was further processed prior to 454 sequencing to enhance yield and sequencing quality. First, the pooled sample was purified using AMPure size exclusion beads (Agencourt, MA, USA). This size-selection is essential for efficient emulsion PCR (emPCR) because small fragments would be preferentially amplified, causing eventual sequence loss by decreasing the overall average read length. Next, the biotin-linked fragments were captured onto streptavidin-coated paramagnetic beads (Invitrogen, CA, USA) and denatured using sodium hydroxide. This procedure was used to purify the fragments containing both adaptors (biotin-A and B). Incorrectly ligated fragments containing B adaptors at both ends failed to bind to beads initially, allowing their removal. After denaturation, those fragments with two biotin-A adaptors remained attached to the beads, leaving the correct constructs (with biotin-A and B) in the supernatant. Note that, because our library was essentially free of incorrect adaptor configurations (e.g., two A adaptors, etc.), this biotin-streptavidin purification step was not actually necessary, but is described here for completeness. Finally, the size distribution of this purified, single-stranded template cDNA (sstcDNA) library was analyzed using an Agilent Bioanalyzer, and the sample was quantified using the Quant-iT OliGreen ssDNA Assay (Invitrogen).

Following cleanup and quantification, the sample was titrated to optimize yield and sequence quality. This procedure was conducted essentially as outlined in the Long Pair-End Library Preparation protocol (Roche, IN, USA) with the following modifications. First, emPCR reactions were performed in triplicate with different amounts of input sstcDNA (0.5, 1, 4, and 16 copies per bead). The emulsions were broken and the DNA Capture beads collected and pooled by sstcDNA concentration. Each pool was then enriched for beads carrying sstcDNA according to the manufacturer's instructions (Roche). The enriched bead samples were then counted using a Z1 Coulter Counter (Beckmann Coulter, CA, USA) to calculate the percent enrichment (i.e., the percent of initial beads that contained sstcDNA). Based on a linear regression of these percent enrichment values against the initial sstcDNA amounts, we calculated the amount of input DNA expected to produce 10–15% enrichment. In our experience, this range of enriched bead recovery maximizes the number of beads containing a single amplified fragment of sstcDNA, while minimizing the number of beads containing multiple fragments. This procedure is essential for maximizing the number of reads produced by 454 sequencing. Based on these analyses, large-scale emPCR was conducted based on the calculated optimum sstcDNA amount, and sequenced using the 454 GS-Flx instrument according to the manufacturers' instructions (Roche).

### Sequence data analysis and assembly

All sequence analyses were conducted using publicly available software and custom Perl scripts that relied heavily upon BioPerl modules [42]. Those bioinformatics scripts, and a detailed protocol describing their use, are available at the authors' website [43].

Adaptor sequences that would prevent correct assembly of the raw sequencing reads were removed following a similar strategy to that used in NCBI's VecScreen tool, based on the known adaptor sequences used in library preparation. Known adaptor sequences were compared against the raw reads using standalone blast (blastn) with permissive settings, to allow detection of weak matches as well as perfect matches (-W 4, -F F, -e 6000). Short matches (≥ 8 bp) were recorded if they fell between two other adaptor matches, or within 10 bp of either end of the read. Longer (≥ 14 bp) matches were required for internal adaptor matches. The adaptor matches so identified, and all sequence distal to those matches, were trimmed from each read using custom scripts.

Most of the trimmed reads (92%) were between 100 and 300 bp in length, but a small proportion fell outside this range. On the assumption that those outlier reads might represent rare sequencing artifacts, we size-selected the cleaned reads to eliminate any reads smaller than 60 bp or larger than 340 bp. Those size thresholds were chosen based on analysis of a preliminary assembly in which all reads within those size ranges failed to assemble, supporting the interpretation that they might represent sequencing artifacts.

The trimmed and size-selected reads were assembled using the Newbler assembly program (Roche). To account for adaptor trimming and sequence quality in this assembly, raw sequence files (SFF format) were modified based on the adaptor trimming information described above, and these modified SFF files processed with Newbler. The assembly also included the publicly available EST *A. millepora *sequences from NCBI (n = 11,829), which had been cleaned based on comparisons with the UniVec database of vectors and adaptor sequences [44]. The overlap settings used for this assembly were 30 bp and 90% identity, with all other parameters set in Newbler at the default values.

### Singleton validation

The inclusion of singleton sequences in our dataset was supported by bioinformatic analysis and experimental validation. To evaluate whether singletons contained unique sequences not found among contigs, singletons were compared with contigs (blastn) and both sequence sets were also compared with the Swiss-Prot database (blastx) to exclude those sequences lacking matches to known proteins. The results from these searches were compared to characterize the set of identifiable genes found among singletons but not contigs. Additional experimental support for retaining singleton sequences was obtained by designing primers specific to each of ten randomly-selected singletons using Primer3 [45]. Using 1 ng of the original intact cDNA as template, each target was amplified separately (35–40 cycles of the profiles described above), and the PCR products evaluated by gel electrophoresis to confirm the presence of each sequence in the original cDNA. Finally, the presence of these singleton sequences in intact cDNA samples was confirmed by cloning and sequencing the resulting PCR products (DNA Core Facility at UT Austin).

### Contig joining and clustering

After assembly of short cDNA sequences, gaps can remain due to uneven sampling across the transcript or the presence of regions that are difficult for the 454 sequencing technique to process (e.g., homopolymer stretches: [15]). To account for any gaps remaining between contigs corresponding to different parts of the same transcript, assembled sequences were joined according to shared similarity with known proteins. Using blastx, the assembled sequences were first compared with the well-annotated Swiss-Prot database, and those that lacked matches were subsequently compared to the larger nr protein database. The best match, with a significance threshold of e ≤ 10^-4^, was recorded for each query sequence. For each of these top matches (protein sequences), all queries for which that protein was the best match were considered. If all queries matched to different, non-overlapping regions of the same protein, they were considered to represent different portions of a single transcript. In these cases, the queries (assembled sequences) were concatenated together in the proper order and strand to recapitulate the original transcript. Gaps within these constructs, representing regions of that transcript not sampled in our libraries, were denoted by strings of 'X' characters to mark the junction between each pair of joined sequences. For those cases where two or more queries matched to overlapping regions of the same protein, all assembled sequences that matched that protein were considered to be possible paralogs and were retained as individual sequences.

### Validation of contig joining

The procedure used to join contigs into scaffolds was validated by PCR using primers corresponding to scaffold sequences. For each of 10 randomly-selected scaffolds, a forward primer was designed based on one of the constituent sequences, and a reverse primer designed based on the other. Amplification with these primer pairs allowed us to test whether, in the intact cDNA, the two primer binding sites were found on the same cDNA molecule, providing experimental validation of the contig joining procedure. PCR was carried out as described above (but for only 20 cycles), and the products were evaluated by gel electrophoresis then cloned and sequenced at the DNA Core Facility at UT Austin.

### Sequence clustering

Assembly of transcriptome sequences can produce a number of contigs that is larger than the number of expressed genes, reflecting redundancy among the assembled sequences (i.e., more than one sequence per gene). Two complementary approaches were employed to characterize the redundancy remaining after assembly and contig joining. First, the sequences with significant protein sequence blast hits were clustered based on sequence similarity shared between those blast hits. Protein sequences were extracted from NCBI protein sequence databases (Swiss-Prot and nr), based on the top hit accession numbers from the blast report, and clustered using the Blastclust 2.2.15 program from NCBI [46]. This approach assembles single-linkage clusters that include contigs corresponding to different domains of the same gene, as well as paralogs of that gene. A disadvantage of this strategy is that because it depends on protein sequence matches, the sequences lacking protein matches cannot be clustered. To reduce redundancy among those non-matching sequences, the complete set of assembled sequences was clustered based on nucleotide sequence similarity in parallel. The precluster and estcluster programs from the ESTate software package were used for this analysis [47]. Finally, the union of these two sets of clusters was obtained by adding all nucleotide sequence relatives of each protein cluster member to that cluster. Individual sequences were annotated with cluster membership information and maintained as separate sequences for further annotation.

### Annotation

Putative gene names, protein domains, and Gene Ontology terms were assigned to all assembled sequences that shared sequence similarity with previously identified genes annotated with those details. Sequence comparisons and annotation were accomplished using standalone blast from NCBI [46], WU-Blast 2.0 (04-May-2006) [48], and custom Perl scripts. For gene name annotation, the assembled sequences were compared against Swiss-Prot and nr protein sequence databases using blastx with a significance threshold of e ≤ 10^-4^. Gene names were assigned to each assembled sequence based on the best blast hit annotated with a gene name, as determined by parsing the GenBank record features 'CDS' and 'Protein' for that blast hit. Blast hits annotated with uninformative terms were encountered (e.g., 'unknown', 'uncharacterized', or 'hypothetical') were skipped in this analysis. For computational speed, the search was limited to the first 10 significant hits for each query.

Different genes can share certain conserved domains, reflecting a level of sequence similarity not accounted for by simple gene name annotation. To identify conserved protein domains in our library, the assembled sequences were compared with the CDD database of conserved domains [49]. These searches were based on reverse position specific blast (e-value threshold: 10^-4^), a blast-like algorithm for comparing newly-identified sequences to a set of known profiles [50]. Each sequence that matched a conserved protein domain was annotated with that domain identifier and a description of the domain corresponding to the best rps-blast match for that query.

To annotate assembled sequences with Gene Ontology (GO) terms describing biological processes [51], molecular functions, and cellular components, sequences were compared against the UniProt-TrEMBL database of protein sequences [52] using WU-blast. This database was chosen because it has been extensively annotated with GO terms [53]. Each query sequence was assigned GO terms based on those associated with the top blast match for that query, using custom Perl scripts to compare the blast results and the table of annotations. Because the Gene Ontology is hierarchical in structure, the GO terms annotated here can be traced back to one or more parent terms at a given level of detail. For example, inflammatory response (a level 5 term) is a 'child' of the more general level 4 term, response to wounding. Custom scripts were used to compare the annotated GO terms with the full set of parent-child relationships to map these annotations to various levels. To demonstrate the utility of these annotations, we used these GO mappings to search for genes associated with processes and molecular functions known to be involved with stress responses in corals.

Finally, the annotated sequences were compared with the RepBase database of transposable elements (TE) [54]. Assembled sequences were queried against this database using tblastx, and those showing significant matches to known TE records were noted. This allows sequences showing similarity to TEs to be excluded from further analysis when required.

The cleaned sequencing reads produced in this study have been deposited in NCBI's SRA database, along with the assembly output (accession: SRA003728). The assembled and annotated sequences have been deposited in the TSA database (accessions: EZ000637–EZ048765). The complete set of sequences is available as a flat file from the authors' website [43], and can be searched online at SymBioSys, a sequence database that focuses on cnidarians and their algal symbionts [55].

### Comparison with *Nematostella vectensis*

Because no genome sequence is available for *A. millepora *or any other scleractinian coral, the sequences generated in the current study were compared with the predicted protein sequences from the most closely related organism with a fully sequence genome: the anemone *Nematostella vectensis *[25]. The *A. millepora *sequences were compared with these protein sequences [24] using blastx with a significance threshold of e ≤ 10^-4^. A similar comparison between the *N. vectensis *genome assembly and the *A. millepora *sequences was conducted using tblastx, to detect any coral sequences with anemone orthologs that had been missed by gene prediction algorithms. Comparisons between the results from these searches and the annotation of the *A. millepora *sequences as described above were used to identify genes present in the coral but not the anemone. Reciprocal comparisons of anemone protein sequences against assembled coral sequences (tblastn) allowed identification of orthologous genes. To correct for different database sizes in these searches (~100,000 coral sequences, ~27,000 anemone sequences), e-values for reciprocal searches were adjusted to a standard database size (equal to the nr protein database). Pairs of orthologous genes in coral and anemone were identified based on reciprocal best matches with adjusted e-values ≤ 10^-4 ^or better.

### SNP detection and validation

Potential SNPs were detected using the QualitySNP program [36], which employs a haplotype-based strategy to detect reliable SNPs without requiring sequence traces, quality scores, or genomic sequence data. SNP identification was accomplished through a separate procedure from the main annotation pipeline, consisting of the following steps. First, all 634,070 reads were assembled using the CAP3 program [56]. Next, the contig alignments from this assembly were evaluated to select clusters containing at least 5 reads. Within these clusters, SNPs were identified based on the filters described in [36].

Twenty predicted SNPs corresponding to 18 genes were chosen for a validation procedure involving primer design, PCR amplification and Sanger sequencing. The 18 contigs containing these SNPs were selected based on sequence similarity with genes presumed to be involved in stress responses in corals. Primers were designed based on several principles as described in [41] so that all PCR amplifications could be accomplished at the same annealing temperature. PCR reactions were each set up in a 10 μl volume containing 5 ng of the amplified intact cDNA (i.e., prior to sonication), 0.1 μM each primer, 2 mM MgCl_2_, 0.3 mM dNTP, 1× PCR buffer and 0.5 U *Taq *DNA polymerase (New England BioLabs). The profile consisted of an initial denaturation step at 94°C for 3 min; this was followed by 30 cycles of 94°C for 30 s, 60°C for 30 s, and 72°C for 30 s; and a final extension step at 72°C for 10 min. After evaluating their molecular weight and specificity based on gel electrophoresis, PCR products were ligated to pGEM-T vector (Promega) and transformed into Top10 *E. coli *competent cells (Invitrogen). Based on single colonies selected from these transformations, cloned inserts were amplified by colony PCR using M13-27 and M13-41 primers. The resulting PCR products were cleaned with ExoSAP-IT (USB, OH, USA) and sequenced at the DNA Core Facility at UT Austin, using M13-27 as the sequencing primer. Two to twenty colonies were sequenced for each predicted SNP, allowing confirmation (if both alleles were observed) or rejection (if only a single allele was found) of those predictions.

## Authors' contributions

EM drafted the manuscript, created the bioinformatics scripts, conducted sequence analysis and functional annotation, and participated in sample collection and library preparation. GVA participated in library preparation. SW conducted the SNP analysis and validation. JBC and JC conducted library titration, 454 sequencing, and the Newbler assembly. BLW and DA were instrumental in sample collection and logistical field support. MVM conceived the study and library preparation methods, and participated in sample collection. All authors read and approved the final manuscript.

## Supplementary Material

Additional file 1 **Validation of singleton sequences and contig joining procedure by PCR amplification**. This document contains gel photographs of the PCR products obtained from validation of randomly-selected scaffold and singleton sequences. Click here for file

Additional file 2 **Effects of sequence length on the proportion of sequences for which significant matches were found**. This PDF file contains a bar plot summarizing the effects of sequence length on the proportion of sequences for which significant blast matches were found. Click here for file

Additional file 3 **Validation of predicted SNPs by PCR and Sanger sequencing**. The table in this document shows the primer sequences used to amplify and sequence each of the 20 SNPs selected for validation, as well as the different alleles detected for each. Click here for file

Additional file 4 **Detailed protocol for preparing cDNA for 454 transcriptome sequencing**. This document gives a step-by-step protocol for preparing cDNA for 454 sequencing. Click here for file

## References

[B1] Moberg F, Folke C (1999). Ecological goods and services of coral reef ecosystems. Ecological Economics.

[B2] Harvell CD, Jordan-Dahlgren E, Merkel S, Rosenberg E, Raymundo L, Smith G, Weil E, Willis B (2007). Coral disease, environmental drivers, and the balance between coral and microbial associates. Oceanography.

[B3] Hoegh-Guldberg O, Mumby PJ, Hooten AJ, Steneck RS, Greenfield P, Gomez E, Harvell CD, Sale PF, Edwards AJ, Caldeira K (2007). Coral Reefs Under Rapid Climate Change and Ocean Acidification. Science.

[B4] Hughes TP, Baird AH, Bellwood DR, Card M, Connolly SR, Folke C, Grosberg R, Hoegh-Guldberg O, Jackson JBC, Kleypas J (2003). Climate change, human impacts, and the resilience of coral reefs. Science.

[B5] Donner SD, Skirving WJ, Little CM, Oppenheimer M, Hoegh-Guldberg O (2005). Global assessment of coral bleaching and required rates of adaptation under climate change. Global Change Biology.

[B6] Douglas AE (2003). Coral bleaching – how and why?. Marine Pollution Bulletin.

[B7] Baums IB (2008). A restoration genetics guide for coral reef conservation. Mol Ecol.

[B8] Day T, Nagel L, van Oppen MJ, Caley MJ (2008). Factors affecting the evolution of bleaching resistance in corals. Am Nat.

[B9] Van Oppen MJH, Gates RD (2006). Conservation genetics and the resilience of reef-building corals. Molecular Ecology.

[B10] Kortschak RD, Samuel G, Saint R, Miller DJ (2003). EST analysis of the Cnidarian Acropora millepora reveals extensive gene loss and rapid sequence divergence in the model invertebrates. Current Biology.

[B11] Schwarz JA, Brokstein PB, Voolstra C, Terry AY, Miller DJ, Szmant AM, Coffroth MA, Medina M (2008). Coral life history and symbiosis: Functional genomic resources for two reef building Caribbean corals, Acropora palmata and Montastraea faveolata. BMC Genomics.

[B12] Technau U, Rudd S, Maxwell P, Gordon PMK, Saina M, Grasso LC, Hayward DC, Sensen CW, Saint R, Holstein TW (2005). Maintenance of ancestral complexity and non-metazoan genes in two basal cnidarians. Trends in Genetics.

[B13] Forêt S, Kassahn KS, Grasso LC, Hayward DC, Iguchi A, Ball EE, Miller DJ (2007). Genomic and microarray approaches to coral reef conservation biology. Coral Reefs.

[B14] Edge SE, Morgan MB, Gleason DF, Snell TW (2005). Development of a coral cDNA array to examine gene expression profiles in Montastraea faveolata exposed to environmental stress. Mar Pollut Bull.

[B15] Margulies M, Egholm M, Altman WE, Attiya S, Bader JS, Bemben LA, Berka J, Braverman MS, Chen YJ, Chen Z (2005). Genome sequencing in microfabricated high-density picolitre reactors. Nature.

[B16] Hudson ME (2008). Sequencing breakthroughs for genomic ecology and evolutionary biology. Molecular Ecology Resources.

[B17] Cheung F, Haas BJ, Goldberg SMD, May GD, Xiao YL, Town CD (2006). Sequencing Medicago truncatula expressed sequenced tags using 454 Life Sciences technology. Bmc Genomics.

[B18] Emrich SJ, Barbazuk WB, Li L, Schnable PS (2007). Gene discovery and annotation using LCM-454 transcriptome sequencing. Genome Research.

[B19] Cheung F, Win J, Lang JM, Hamilton J, Vuong H, Leach JE, Kamoun S, Levesque CA, Tisserat N, Buell CR (2008). Analysis of the Pythium ultimum transcriptome using Sanger and Pyrosequencing approaches. BMC Genomics.

[B20] Mao C, Evans C, Jensen RV, Sobral BWS (2008). Identification of new genes in Sinorhizobium meliloti using the Genome Sequencer FLX system. BMC Microbiology.

[B21] Novaes E, Drost DR, Farmerie WG, Pappas GJ, Grattapaglia D, Sederoff RR, Kirst M (2008). High-throughput gene and SNP discovery in Eucalyptus grandis, an uncharacterized genome. Bmc Genomics.

[B22] Toth AL, Varala K, Newman TC, Miguez FE, Hutchison SK, Willoughby DA, Simons JF, Egholm M, Hunt JH, Hudson ME (2007). Wasp gene expression supports an evolutionary link between maternal behavior and eusociality. Science.

[B23] Vera JC, Wheat CW, Fescemyer HW, Frilander MJ, Crawford DL, Hanski I, Marden JH (2008). Rapid transcriptome characterization for a nonmodel organism using 454 pyrosequencing. Molecular Ecology.

[B24] JGI Nematostella vectensis v1.0. http://genome.jgi-psf.org/Nemve1/Nemve1.download.ftp.html.

[B25] Putnam NH, Srivastava M, Hellsten U, Dirks B, Chapman J, Salamov A, Terry A, Shapiro H, Lindquist E, Kapitonov VV (2007). Sea anemone genome reveals ancestral eumetazoan gene repertoire and genomic organization. Science.

[B26] Samanta MP, Tongprasit W, Istrail S, Cameron RA, Tu Q, Davidson EH, Stolc V (2006). The transcriptome of the sea urchin embryo. Science.

[B27] McKay SJ, Johnsen R, Khattra J, Asano J, Baillie DL, Chan S, Dube N, Fang L, Goszczynski B, Ha E (2003). Gene Expression Profiling of Cells, Tissues, and Developmental Stages of the Nematode C. elegans. Cold Spring Harb Symp Quant Biol.

[B28] Manak JR, Dike S, Sementchenko V, Kapranov P, Biemar F, Long J, Cheng J, Bell I, Ghosh S, Piccolboni A (2006). Biological function of unannotated transcription during the early development of Drosophila melanogaster. Nature Genetics.

[B29] Karp PD, Ouzounis CA, Moore-Kochlacs C, Goldovsky L, Kaipa P, Ahren D, Tsoka S, Darzentas N, Kunin V, Lopez-Bigas N (2005). Expansion of the BioCyc collection of pathway/genome databases to 160 genomes. Nucleic Acids Research.

[B30] Ruepp A, Brauner B, Dunger-Kaltenbach I, Frishman G, Montrone C, Stransky M, Waegele B, Schmidt T, Doudieu ON, Mpflen VS (2008). CORUM: the comprehensive resource of mammalian protein complexes. Nucleic Acids Research.

[B31] Srivastava M, Begovic E, Chapman J, Putnam NH, Hellsten U, Kawashima T, Kuo A, Mitros T, Salamov A, Carpenter ML (2008). The Trichoplax genome and the nature of placozoans. Nature.

[B32] Desalvo MK, Voolstra CR, Sunagawa S, Schwarz JA, Stillman JH, Coffroth MA, Szmant AM, Medina M (2008). Differential gene expression during thermal stress and bleaching in the Caribbean coral Montastraea faveolata. Molecular Ecology.

[B33] Grasso LC, Maindonald J, Rudd S, Hayward DC, Saint R, Miller DJ, Ball EE (2008). Microarray analysis identifies candidate genes for key roles in coral development. BMC Genomics.

[B34] Van Oppen MJH, Underwood J, Muirhead AN, Peplow L (2007). Ten microsatellite loci for the reef-building coral Acropora millepora (Cnidaria, Scleractinia) from the Great Barrier Reef, Australia. Molecular Ecology Notes.

[B35] Barbazuk WB, Emrich SJ, Chen HD, Li L, Schnable PS (2007). SNP discovery via 454 transcriptome sequencing. Plant Journal.

[B36] Tang JF, Vosman B, Voorrips RE, Linden CG Van der, Leunissen JAM (2006). QualitySNP: a pipeline for detecting single nucleotide polymorphisms and insertions/deletions in EST data from diploid and polyploid species. Bmc Bioinformatics.

[B37] Babcock RC, Bull GD, Harrison PL, Heyward AJ, Oliver JK, Wallace CC, Willis BL (1986). Synchronous Spawnings of 105 Scleractinian Coral Species on the Great-Barrier-Reef. Marine Biology.

[B38] Heyward AJ, Negri AP (1999). Natural inducers for coral larval metamorphosis. Coral Reefs.

[B39] Iwao K, Fujisawa T, Hatta M (2002). A cnidarian neuropeptide of the GLWamide family induces metamorphosis of reef-building corals in the genus Acropora. Coral Reefs.

[B40] Matz M, Shagin D, Bogdanova E, Britanova O, Lukyanov S, Diatchenko L, Chenchik A (1999). Amplification of cDNA ends based on template-switching effect and step-out PCR. Nucleic Acids Research.

[B41] Matz MV (2002). Amplification of Representative cDNA Samples from Microscopic Amounts of Invertebrate Tissue to Search for New Genes. Methods Mol Biol.

[B42] Stajich JE, Block D, Boulez K, Brenner SE, Chervitz SA, Dagdigian C, Fuellen G, Gilbert JGR, Korf I, Lapp H (2002). The bioperl toolkit: Perl modules for the life sciences. Genome Research.

[B43] Matz Laboratory Website. http://www.bio.utexas.edu/research/matz_lab/matzlab/Welcome.html.

[B44] The UniVec Database. ftp://ftp.ncbi.nih.gov/pub/UniVec/.

[B45] Primer3. http://primer3.sourceforge.net/.

[B46] NCBI Blast Tools. ftp://ftp.ncbi.nih.gov/blast/executables/release/2.2.15/.

[B47] ESTate. http://www.ebi.ac.uk/~guy/estate/.

[B48] Washington University Blast Archives. http://blast.wustl.edu.

[B49] NCBI Conserved Domain Database. ftp://ftp.ncbi.nih.gov/pub/mmdb/cdd/little_endian/.

[B50] Marchler-Bauer A, Panchenko AR, Shoemaker BA, Thiessen PA, Geer LY, Bryant SH (2002). CDD: a database of conserved domain alignments with links to domain three-dimensional structure. Nucleic Acids Research.

[B51] Ashburner M, Ball CA, Blake JA, Botstein D, Butler H, Cherry JM, Davis AP, Dolinski K, Dwight SS, Eppig JT (2000). Gene Ontology: tool for the unification of biology. Nature Genetics.

[B52] UniProt-TrEMBL. http://www.ebi.ac.uk/trembl/FTP/ftp.html.

[B53] The Gene Ontology. http://www.geneontology.org/GO.downloads.shtml.

[B54] RepBase. http://www.girinst.org/server/RepBase/.

[B55] SymBioSys. http://sequoia.ucmerced.edu/SymBioSys/index.php.

[B56] Huang XQ, Madan A (1999). CAP3: A DNA sequence assembly program. Genome Research.

