# Supplementary material for: Sequencing and de novo analysis of a coral larval transcriptome using 454 GSFlx
Source: BMC Genomics. 2009 May 12;10:219. doi: 10.1186/1471-2164-10-219 (PMC2689275; doi:10.1186/1471-2164-10-219)
Supplement: Additional file 1 — Validation of singleton sequences and contig joining procedure by PCR amplification. This document contains gel photographs of the PCR products obtained from validation of randomly-selected scaffold and singleton sequences. [file 1471-2164-10-219-S1.doc]

**Additional File 1**


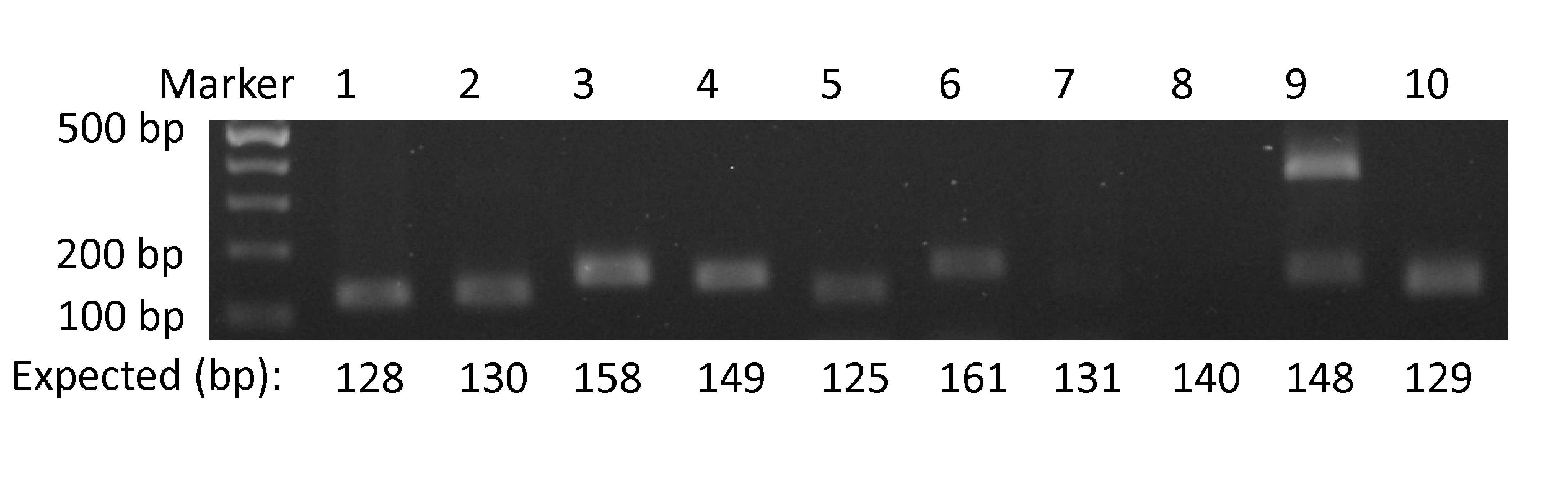


**Validation of singleton sequences.**

PCR products for 10 randomly-selected singleton sequences are shown on an ethidium-bromide stained agarose gel. The expected molecular weights based on positions of primers within each target are shown below each lane.

**
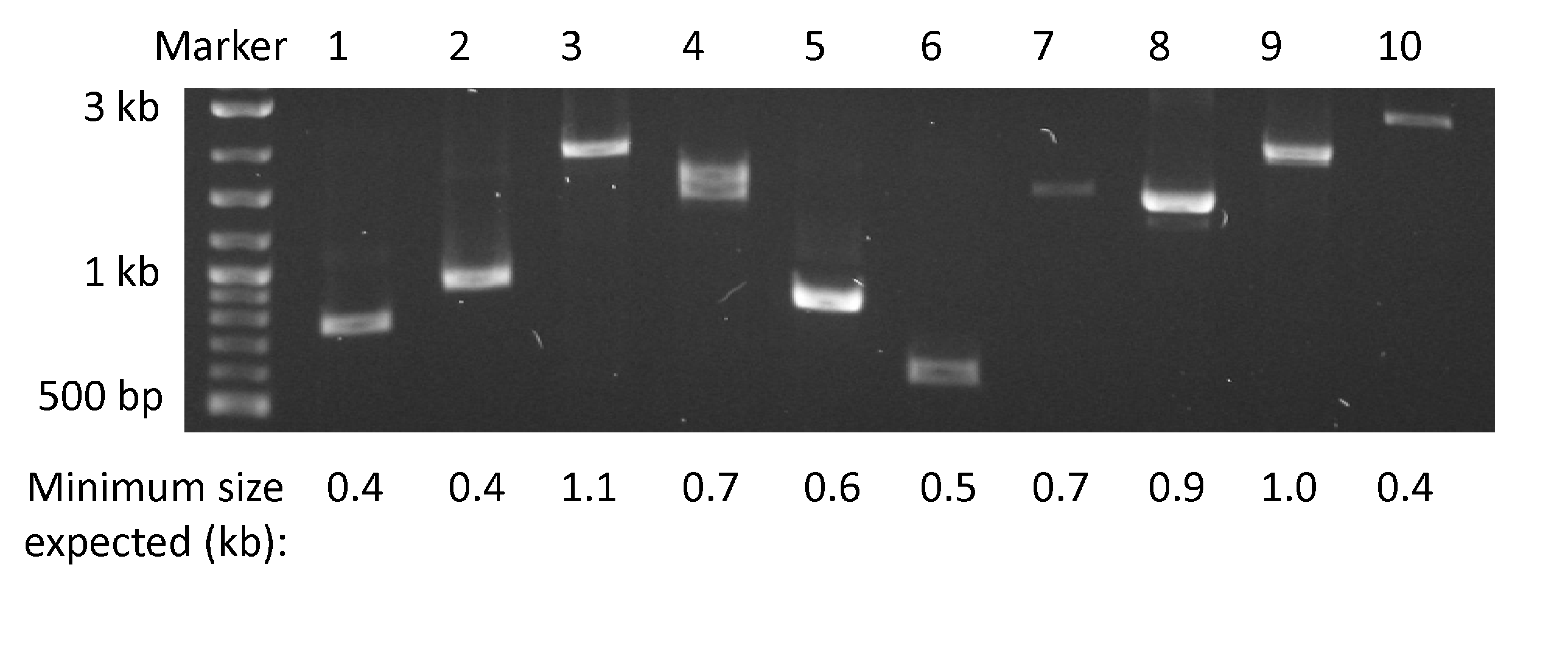
 Validation of scaffolds.**

PCR products for 10 randomly-selected scaffolds are shown on an ethidium-bromide stained agarose gel. Because scaffolds include gaps of unknown size, the minimum expected molecular weights are shown, based on the positions of primers within scaffolds.
