# Supplementary figures and images for: Sequencing and de novo analysis of a coral larval transcriptome using 454 GSFlx
Source: BMC Genomics. 2009 May 12;10:219. doi: 10.1186/1471-2164-10-219 (PMC2689275; doi:10.1186/1471-2164-10-219)

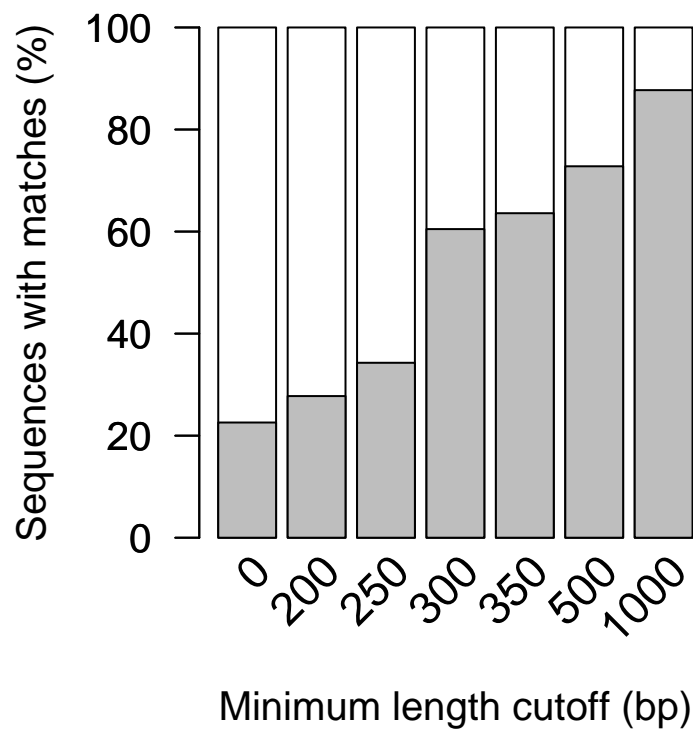

Supplement: Additional file 2 — Effects of sequence length on the proportion of sequences for which significant matches were found. This PDF file contains a bar plot summarizing the effects of sequence length on the proportion of sequences for which significant blast matches were found. [file 1471-2164-10-219-S2.pdf]
