# Supplementary material for: Sequencing and de novo analysis of a coral larval transcriptome using 454 GSFlx
Source: BMC Genomics. 2009 May 12;10:219. doi: 10.1186/1471-2164-10-219 (PMC2689275; doi:10.1186/1471-2164-10-219)
Supplement: Additional file 3 — Validation of predicted SNPs by PCR and Sanger sequencing. The table in this document shows the primer sequences used to amplify and sequence each of the 20 SNPs selected for validation, as well as the different alleles detected for each. [file 1471-2164-10-219-S3.doc]

| Additional File 3 - Validation of predicted SNPs by PCR and re-sequencing | | |
| --- | --- | --- |
| Sequence (putative identity) | SNP | Primer sequences |
| 1. Contig6072 | 440A/G | F: GGGTGGAGTATTCACCAAGCT |
| (Hsp70) |  | R: TGTTTGTCCATCTGCTGCTGTA |
|  | **929T/C** | F: CTGCTGCCTCTCTGAGTCTT |
|  |  | R: ACTGAATTCAAGCGGGAGAGT |
| 2. Contig8737 | *259T/C* | F: CTGGCAGAGATCTCACTGACT |
| (Actin) |  | R: AATCTCACGCTCAGCAGTGGT |
| 3. Contig14848 | **418T/C** | F: CTCATCACTAACTTTTCGTGCAT |
| (Ubiquitin-like protein 3) |  | R: CAACAAAAGAGAACTCCTCTACT |
| 4. Contig16407 | *654A/T* | F: TATTCCAGCAGATATCTCTCGA |
| (14-3-3 protein) |  | R: GCAGAAACACGAAGACAATCCA |
| 5. Contig16728 | **211T/C** | F: AGTCGGTGACATCTTCTTGCAA |
| (Thioredoxin) |  | R: TTGACAATTTCTTGAAGGAGGCT |
| 6. Contig16774 | **501T/C** | F: GAGGACATCTCAATCACAGCAT |
| (Mn superoxide dismutase) |  | R: GATAGCATCCATTAATCCACCTA |
|  | **791A/C** | F: CTGGAAGTTCATGCTTAGCCTT |
|  |  | R: AATGTTGTGCCGTAGTGTTCGA |
| 7. Contig20421 | **239T/G** | F: CGAAACGTGGGTTGTAAGTTCTA |
| (Galaxin) |  | R: GAAAAGGATTTCGACGTTGACGA |
| 8. Contig25861 | *543T/C* | F: GGAGTCCATTTTTCAGGGATGT |
| (Ras-homology protein 1) |  | R: TGTCCTACCCTGATACAGATGT |
| 9. Contig26385 | *345A/G* | F: CTTCTGAGCCATTGACTGCCA |
| (Glutathione peroxidase) |  | R: TCCCTGAAGTATGTGCGTCCT |
| 10. Contig26792 | **293A/G** | F: TCCAGAATTCGATATCTTCTCCA |
| (Complement component C3) |  | R: ACGAAGATGATAAGTCCCTTACT |
| 11. Contig35180 | **523T/G** | F: TCCAGGTTCCATCTTTGACGA |
| (Beta gamma crystallin) |  | R: GTAGTGGCGCCACAAAAGACT |
| 12. Contig45302 | *682T/C* | F: AGAATCCACCTACATTTGGTAGT |
| (Benzodiazepine receptor) |  | R: AATCTAAGGTCGGGGGCTGAA |
| 13. Contig50281 | **478A/G** | F: GGAAGATGCAGGTGTTTACGAT |
| (Arginine kinase) |  | R: CCATTATACATGTCTTGGACACA |
| 14. Contig52394 | **280A/G** | F: TGTCCTTTAGTGGTGTTGATGA |
| (Hsp60) |  | R: GACCCTACAGGATGAATTGGA |
| 15. Contig56996 | *317T/G* | F: CCGCAAGCTGGGTTCAATGA |
| (Ubiquitin) |  | R: TACAACATCCAGAAAGAGTCCA |
| 16. Contig60613 | **230T/C** | F: AGTATGTTGCTGAGAATGTTGCT |
| (Coatomer) |  | R: ATCAAAAGCATCCTCTACACCAT |
| 17. Contig60937 | **139A/C** | F: GGTCAAGAGCTGACAGTCACT |
| (Small heat shock protein) |  | R: AAGGAGGATACGGCGATGGT |
| 18. Contig63538 | **709C/G** | F: CAATGCAGCATCAACTGCATCT |
| (Ligand of numbX2) |  | R: GGACGGTAACAAAGACATCCAA |
| 20 SNPs were selected for validation by searching among QualitySNP contigs for similarity  to 18 known genes thought to be involved in coral stress responses. The 14 validated SNPs  are shown in bold; 6 SNPs that were not confirmed are shown in italics. | | |
