# Supplementary material for: Sequencing and de novo analysis of a coral larval transcriptome using 454 GSFlx
Source: BMC Genomics. 2009 May 12;10:219. doi: 10.1186/1471-2164-10-219 (PMC2689275; doi:10.1186/1471-2164-10-219)
Supplement: Additional file 4 — Detailed protocol for preparing cDNA for 454 transcriptome sequencing. This document gives a step-by-step protocol for preparing cDNA for 454 sequencing. [file 1471-2164-10-219-S4.doc]

Additional File 4 - Preparation of cDNA samples for *de novo* transcriptome sequencing with 454 GS-Flx^TM^.

**Note**: the version of the protocol shown here includes minor modifications from that used for preparing coral larval cDNA. These modifications improve reproducibility and further reduce the occurrence of adaptor concatenation. The improved protocol as described here is recommended for transcriptome analysis in other emerging model organisms.

***Overview***

The initial cDNA is produced using the SMART^TM^ PCR cDNA Synthesis Kit (Clontech, 634902), but with a different cDNA synthesis primer:

Cap-TRSA-CV (first strand cDNA synthesis primer):

5’- AAGCAGTGGTATCAACGCAGAGT CGCAGTCGGTACTTTTTTCTTTTTTV – 3’

(“cap” primer sequence) (“broken chain” polyT)

The purpose of the “broken chain” T-primer is to reduce read artifacts during 454 pyrosequencing, which may get thrown out of calibration by too strong of a light signal produced from a long mononucleotide stretch (such as polyT or polyA).

The cDNA is then:

- [optionally] normalized using Trimmer-Direct Kit (Evrogen, NK002) and re-amplified using cap primer
- nebulized or sonicated to an average fragment size of 350-400 bp
- end-polished (by incubation with a DNA polymerase and dNTPs in appropriate buffer) and ligated to the mixture of “A+” and “B+” adapters.

Each of these adapters is an equimolar mixture of two oligos (typically, 1 µM each in the working concentration): a long one that actually gets ligated at its 3’ end, and a short one that complements to the 3’ end of the longer one to mimic the double-stranded blunt end for the ligase enzyme. The short oligo is not ligated since it lacks a 5’-phosphate.

B+ adapter:

Long oligo: 5’-GCCTTGCCAGCCCGCTCAG ACGAGCGGCCA-3’

(B primer sequence) (suppression tag)

Short oligo: 5’-TGGCCGCTCGT-3’

A+ adapter:

Long oligo: 5’-GCCTCCCTCGCGCCATCAG CCGCGCAGGT-3’

(A primer sequence) (suppression tag)

Short oligo: 5’-ACCTGCGCGG-3’

It is important to note that the adapters only get ligated to the “new” 5’ ends formed as a result of fragmentation/polishing, since the original 5’ termini correspond to the incorporated “cap” primer used for amplification and lack 5’ phosphates.

The library is then amplified using a mixture of four primers. The primers used in this reaction include the standard 454 A and B primers (0.2 µM); these sequences are incorporated into the ligated adaptors shown above. In addition, two long “step-out” primers are used (“A+TRSAC” and “A+halfswitch”) at the lower concentration of 0.005 µM, to incorporate the A+ sequence at each of the original cDNA termini:

A+halfswitch primer:

5’-GCCTCCCTCGCGCCATCAG CCGCGCAGGTAC GTATCAACGCAGAGTACGCGG-3’

(A primer sequence) (suppression tag) (sequence of the 3’-portion of the

template-switch oligo)

A+TRSAC:

5’-GCCTCCCTCGCGCCATCAG CCGCGCAGGT CGCAGTCGGTACTTTTTTCTTTTTT

(A primer sequence) (suppression tag) (sequence of the 3’-portion of the

“broken chain” cDNA synthesis primer)

During this amplification “suppression tags” invoke a PCR suppression effect for the fragments that end up flanked by the same kind of adapter, which results in exclusive amplification of the fragments flanked by both A and B primers. In these fragments B primer is found only on the “inside” of the original cDNA sequence (i.e., fragmentation points introduced during sonication or nebulization) while A pimer can be either inside (by virtue of adaptor ligation) or “outside”, i.e. flanking the original cDNA termini (by virtue of step-out amplification).

The A primer can be biotinylated, so the PCR product can be bound to streptavidin beads. The B primer is used for sequencing. The suppression tag of B primer can be variable and used as a barcode to discriminate samples sequenced simultaneously in the same 454 plate.


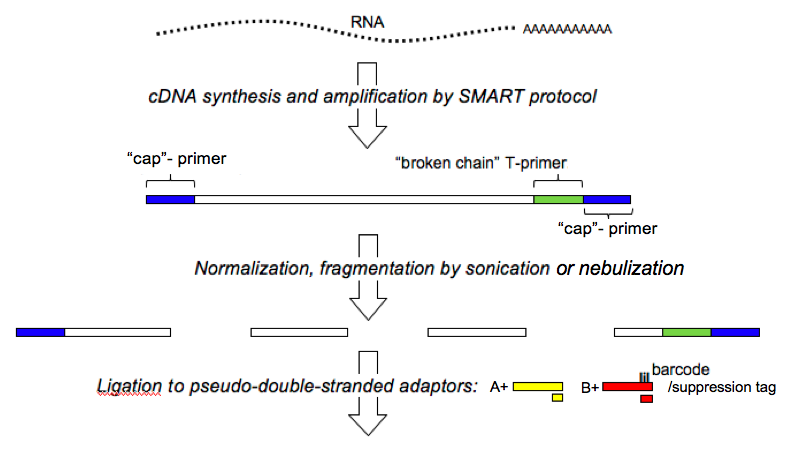


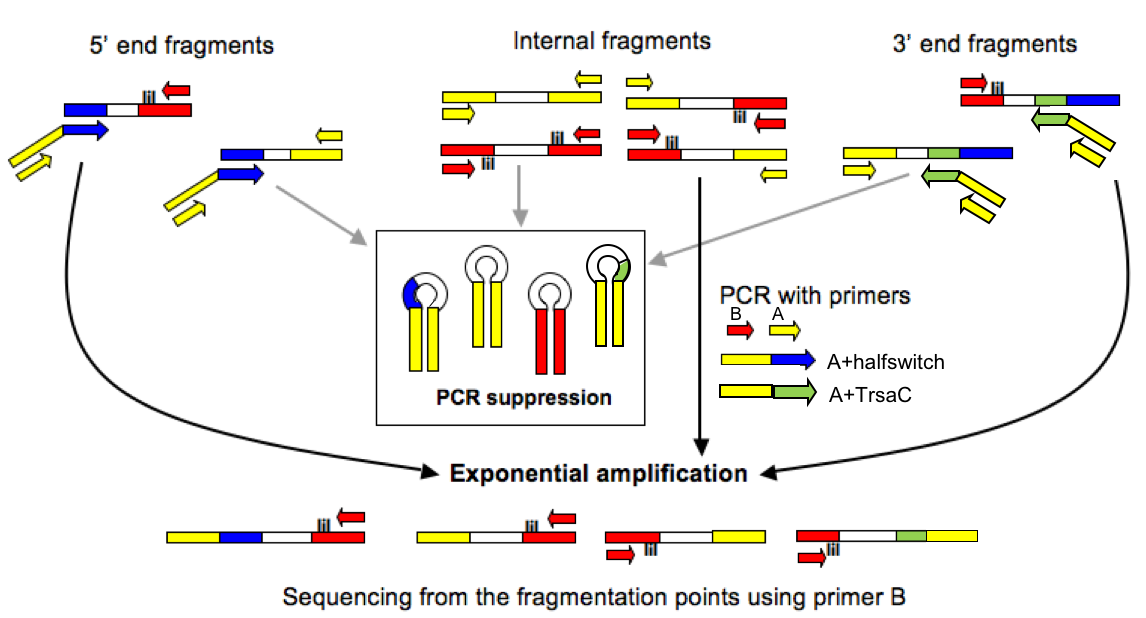


***Detailed protocol***

1. **RNA template preparation.** *These steps are recommended but may not be necessary,*

*depending on your protocol of choice for isolating total RNA.*

1. Begin with about 2-10 µg RNA from the organism of your choice
2. Precipitate RNA by adding 1 volume 13.3 M LiCl, incubating 30 minutes at -20°C, and centrifuging 20 minutes at 16g at room temperature.
3. Rinse RNA pellets briefly with 80% ethanol (don’t centrifuge), air dry at room temperature, and dissolve pellets in EB (10 mM Tris, pH 8.0).
4. Analyze RNA on a gel to evaluate integrity.
5. **First-strand cDNA synthesis** *(At this and the next stage, follow Clontech’s SMART^TM^ PCR cDNA Synthesis protocol, but replace the cDNA synthesis primer with Cap-TRSA-CV)*
6. Combine 4 µl RNA (for a total of 1000 ng RNA) with 1 µl 10 µM Cap-TRSA-CV primer. Incubate 3 minutes at 65°C, then chill on ice.
7. To the above tube, add a premixed solution containing the following:

2 µl 5X first-strand synthesis buffer

0.5 µl 10 mM dNTP

1 µl 0.1 M DTT

1 µl 10 µM template-switch primer (provided in Clontech’s kit)

1 µl Superscript II reverse transcriptase (Invitrogen, 18064-014)

1. Incubate at 42°C for 1 hour.
2. Terminate the reaction by incubating at 65°C for 3 minutes, then return tube to ice.
3. Dilute 5-fold in water to minimize carryover of primers into subsequent reactions.
4. **cDNA amplification**
   1. For each first-strand-cDNA sample, set up 12 PCR reactions (30 µl each):

1 µl diluted FS-cDNA (from step 2e)

23.25 µl H_2_O

3 µl 10X PCR buffer

0.75 µl 10 mM dNTP

1.4 µl 10 µM cDNA amplification primer (provided in Clontech’s kit)

0.6 µl Advantage2 polymerase (Clontech)

- 1. Amplify using the following profile:

94°C for 5 minutes;

(94°C for 40 seconds, 65°C for 1 minute, 72°C for 6 minutes) x (15-19) cycles depending on the sample

After PCR, hold product at room temperature, not on ice.

- 1. Evaluate PCR product by loading 3 µl on a gel and visualizing with ethidium bromide. There should be a **faintly** visible smear with some bands, with the majority of product falling between 500 and 3000 bp in length. Add 3 more cycles if there is nothing visible on the gel, then evaluate again. If the product is not amplified in 20 cycles, something is wrong - start over from the cDNA synthesis step. *NOTE: the total amount of cDNA product per tube should not exceed 200 ng, which means that the smear on the agarose gel (20 ng per lane) should be very faint. Make sure you don’t over-amplify cDNA beyond that*.
  2. To maximize the amount of PCR product that is double-stranded, “chase” the reactions by adding the original amount of primer again (1.4 µl of 10 µM cDNA amplification primer) and cycling with the following profile:

78°C for 1 minute, 65°C for 1 minute, 72°C for 7 minutes.

- 1. Combine together 12 separate reactions prepared from each first-strand-cDNA sample, and purify this PCR product on a column (we use Qiagen Qiaquick PCR Purification kit). Elute the final sample in 50 µl of EB (10 mM tris-HCl pH 8.0). Measure the concentration of DNA using Nanodrop spectrophotometer or any other appropriate method; there should be at least 2 µg of DNA in total.
  2. EtOH precipitate the product to concentrate (i.e. if the resulting concentration is less than 2 µg in 12 µl).

5 µl (0.1X volume) 3M NaAcetate pH 4.8-5.2

125 µl (2.5X volume) 96-100% EtOH

hold 20-30 minutes at -20°C

Spin 20 minutes at maximum speed at 4°C, rinse with 70% EtOH, air dry, dissolve in

appropriate volume of milliQ water to achieve a concentration of 2 µg in 12 µl.

1. **Normalization.** *The Trimmer-Direct Kit from Evrogen is used essentially according to the manufacturer’s instructions, as summarized below.*
   1. Prepare a hybridization master mix by combining:

2 µg cDNA from step 3f in ≤ 12 µl volume

4 µl 4X hybridization buffer

H_2_O to a total volume of 16 µl

(Note that final cDNA concentration = 125 ng µl^-1^)

- 1. Aliquot this out into 4 individual PCR tubes (4 µl each) and overlay each with a drop of sterile mineral oil; centrifuge briefly to collect liquid and separate phases.
  2. Using a thermal cycler, incubate at 98°C for 2 minutes, then at 68°C for 5 hours, then proceed immediately to the next step.
  3. Near the end of the hybridization period (step 4d), warm the DSN master buffer (Trimmer kit) to 68°C.
  4. Prepare ½ and ¼ strength dilutions of the double-strand specific nuclease (DSN) using DSN storage buffer as the diluent; store on ice until ready to use.
  5. At the end of the hybridization period, add 5 µl preheated master buffer to each tube. Spin briefly in a bench-top centrifuge and return immediately to the thermal cycler. It is important to maintain the temperature at 68°C during this period, so minimize time spent out of the thermal cycler (no more than a few seconds).
  6. To the four tubes from step 4c, add the following, while maintaining temperature:

Tube Add

A 1 µl un-diluted DSN enzyme

B 1 µl ½ dilution DSN enzyme

C 1 µl ¼ dilution DSN enzyme

D 1 µl DSN storage buffer (diluent)

- 1. Incubate at 68°C for 25 minutes.
  2. Add 10 µl of DSN stop solution (Trimmer kit) to each tube, mix well, and spin briefly to collect contents.
  3. Incubate at 68°C an additional 5 minutes.
  4. Add 20 µl H_2_O to each tube then store at -20°C or proceed with next steps.

1. **Amplification of normalized cDNA**
   1. Set up 4 separate PCR reactions, each containing:

1 µl diluted normalized cDNA (from step 4l), one PCR reaction per DSN treatment

23.25 µl H_2_O

3 µl 10X PCR buffer

0.75 µl 10 mM dNTP

1.4 µl 10 µM cDNA amplification primer (provided in Clontech’s kit)

0.6 µl Advantage2 polymerase (Clontech)

- 1. Amplify using the following profile:

94°C for 5 minutes;

(94°C for 40 seconds, 65°C for 1 minute, 72°C for 6 minutes) x 5 cycles

- 1. Remove all tubes from thermal cycler. Remove a 5-µl aliquot from the control tube (corresponding to template tube D, in step 4h) and set this aside.
  2. Amplify the control tube for an additional 2 cycles (total = 7). Remove another 5-µl aliquot and set aside.
  3. Repeat step 5d twice more, producing aliquots from this tube that correspond to 5, 7, 9 and 11 cycles.
  4. Load all aliquots from step 5e on a gel to evaluate optimum cycle number *X* as described in the manufacturer’s instructions (for our experiments, *X* = 6).
  5. Return DSN-containing reactions to the thermal cycler and amplify for an additional *N* cycles, where *N* = *X* + 9 - 5 (for our experiments, *X* + 9 - 5 = 10, for 15 cycles total in experimental tubes).
  6. “Chase” all reactions as described in step 3d.
  7. Load 5 µl on a gel to determine which enzyme dilution treatment (1, ½, or ¼) gave the best results, as described in Trimmer kit instructions.
  8. Once both the optimum cycle number (step 5g) and the optimum enzyme treatment (step 5i) have been established, prepare 16 individual 30-µl reactions according to those treatments and repeat steps 5a-i. Again, avoid over-amplifying the cDNA (see note at the step 3c).
  9. Pool the products, purify on a column (e.g., Qiagen Qiaquick), elute in EB, and quantify. Normalized cDNA can be stored at -20°C.

1. **Fragmentation by sonication.** *We prefer sonication to nebulization since it makes it easier to process multiple samples at once, and poses less threat of DNA contamination. We use the sonicator with a “cup horn” attachment: a water-filled cup with a sonicating bottom in which the 1.5 mL tubes may be submerged. Our model is called “ultrasonic liquid processor Sonicator 3000” by Misonix, with cup horn part number 431C.*
   1. Prepare a tube of normalized, amplified, purified cDNA (from step 5k) containing ~ 1-5 µg cDNA in 100 µl. Dilute with EB if required to achieve this concentration (~ 50 ng µl^-1^).
   2. Set aside an aliquot of intact cDNA at this time for later gel analysis.
   3. Set up a sonicator with an ice water bath so that a 1.5-ml centrifuge tube can be partially submerged in the water, with the bottom of the tube resting ~ 1 cm above the cup horn bottom, and the portion of that tube containing liquid fully submerged in the water.
   4. Set the sonicator power at 1.0 – 1.5, corresponding to 18-30W.
   5. Sonication should be done in 30 second “on” bursts, with 30 second “off” rests in between. Note that sonication times are reported here as the sum of all “on” periods during the process.
   6. Sonicate the cDNA for a series of increasing durations, and remove an aliquot at each interval. In our experiments, we choose 1 minute, 3 minutes, 5 minutes and 10 minutes.
   7. After all sonication is complete, load 2-3 µl of each sample (including the original intact cDNA) on a gel to evaluate the molecular weight. Select the treatment that produced a smear ranging from about 300 to about 1000 bp. In our experiments, this is commonly the 3 minute treatment.
   8. Precipitate the fragmented cDNA with ethanol to remove very short oligonucleotides, and dissolve in 20 µl of a suitable buffer (EB or 1X NEB2).
2. **Ligation with adaptors**
   1. Polish the fragmented cDNA to ensure that all ends are blunted, by combining the following in a tube at room temperature:

25 ng fragmented cDNA (from step 6h)

1.25 µl 10X NEB2 buffer

1.25 µl 10X BSA

0.6 µl 10 mM dNTP

0.6 µl T4 DNA polymerase (NEB, M0203S)

0.6 µl Klenow fragment of DNA polymerase I (NEB, M0210S)

H_2_O to final volume = 12.5 µl

- 1. Incubate at room temperature for 1 ½ hours.
  2. Terminate polishing reaction by incubating at 70°C for 15 minutes, then cool to room temperature.
  3. Prepare adaptor A by combining A+ primer and anti-A+ primer at a final concentration of 10 µM each. Repeat for B+1 primer and anti-B+1 primer to prepare adaptor B.
  4. Prepare ligation master mixes at room temperature by combining:

5 µl H_2_O

1.25 µl 10X T4 DNA ligase buffer

2.5 µl 10 µM adaptor A

2.5 µl 10 µM adaptor B

1.25 µl T4 DNA ligase (NEB, M0202L)

- 1. Combine 12.5 µl master mix with 12.5 µl polished cDNA (from step 7c) for a final volume of 25 µl.
  2. Incubate at 12°C overnight.
  3. The following day, dilute ligation 1:5 in H_2_O (total volume 125 µl) and incubate 10 minutes at 65°C, then cool to room temperature – do not store on ice.
  4. Purify on a column (e.g., Qiagen Qiaquick) according to the manufacturer’s instructions, and elute in 30 µl EB.

1. **PCR testing the ligation**
   1. For each 454 cDNA library produced, prepare 4 different PCR reactions, each with a different combination of primers. Including water controls (no-template controls) is recommended. The primer combinations are as follows (final primer concentrations are shown):

Tube Primers

- - - 1. 0.2 µM A
      2. 0.2 µM B
      3. 0.2 µM A, 0.2 µM B
      4. 0.2 µM A, 0.2 µM B, 0.005 µM A+halfswitch, 0.005 µM A+TRSAC
  1. Each PCR reaction is assembled as follows:

3 µl 10X PCR buffer

0.75 µl 10 mM dNTP

1 µl ligation product (from step 7i)

0.6 µl Advantage2 polymerase

Primers as shown above (8a)

H_2_O to final volume of 30 µl

- 1. Amplify these reactions using the following profile:

94°C for 5 minutes;

(94°C for 40 seconds, 65°C for 1 minute, 72°C for 4 minutes) x 17 cycles

- 1. Load 3 µl of these products on a gel; hold remainder at room temperature while the gel runs in case additional cycles are required.
  2. A visible smear ranging from 100-1200 bp should be visible in reaction #4 (all primers), and a slightly smaller and fainter smear in reaction #3 (A+B). None of the other two reactions should produce any product.
  3. If nothing is visible in any lanes, amplify for an additional 2 cycles and repeat the gel analysis.
  4. Repeat step 8f until visible smears are produced to allow determination of optimum cycle number. If more than 17 cycles were required to produce visible smears, try adding more template, rather than performing more PCR cycles. In our experiments, 1 µl of purified ligation product as PCR template and 17 cycles produced visible smears based on loading 3 µl on a gel.

1. **Amplification of a 454-ready sample**
   1. Set up “bulk” amplifications based on the optimum cycle numbers and template volumes determined from the PCR tests above (steps 8a-g). For our experiments, we set up 16 reactions.
   2. Each reaction is assembled as follows:

3 µl 10X PCR buffer

0.75 µl 10 mM dNTP

*X* µl ligation product (determined in steps 8a-g)

0.6 µl 10 µM 5’-biotinylated primer A

0.6 µl 10 µM primer B

0.3 µl 0.5 µM primer A+halfswitch

0.3 µl 0.5 µM primer A+TRSAC

0.6 µl Advantage2 polymerase

H_2_O to final volume of 30 µl

- 1. Amplify the reactions using the following profile:

94°C for 5 minutes;

(94°C for 40 seconds, 65°C 1 minute, 72°C for 4 minutes) X *N* cycles;

where *N* is the optimum cycle number determined (8a-g).

- 1. Load an aliquot on a gel to verify that the reaction amplified as expected.
  2. Chase: add the same amount of B and biotynilated A primers as at the start (8b) and run the following profile:

78°C 1 minute, 65°C 1 minute, 72°C 4 minutes.

- 1. PCR purify using a column (e.g., Qiagen Qiaquick), elute in 50 µl EB, and quantify.
  2. If you have ≥ 5 µg of material, the library is now ready for 454 sequencing.

NOTE: Before committing to the 454 process, we recommend verifying that the final cDNA sample is free from artifacts. Ligate an aliquot of it into any PCR-cloning vector (such as pGEM-T, Promega) and sequence 10-20 randomly picked clones using the standard Sanger technique.

# 
